# Diverse Subpopulations of Reactive Astrocytes Following Chronic Toxoplasma Infection

**DOI:** 10.1002/glia.70053

**Published:** 2025-07-09

**Authors:** Zoe A. Figueroa, Jose L. Martin, Arzu Ulu, William Agnew‐Svoboda, Teresa Ubina, Martin M. Riccomagno, Todd A. Fiacco, Emma H. Wilson

**Affiliations:** ^1^ Division of Biomedical Sciences, School of Medicine University of California Riverside California USA; ^2^ Department Molecular, Cell, and Systems Biology University of California Riverside California USA

**Keywords:** angiogenesis, astrogliosis, glutamate transporters, lipocalin‐2 (Lcn2), neuroinflammation, *Toxoplasma gondii*, transthyretin (Ttr)

## Abstract

Astrocytes provide physical and metabolic support for neurons, regulate the blood–brain barrier, and react to injury, infection, and disease. When astrocytes become reactive, maintenance of the inflammatory state and its functional implications throughout chronic neuroinflammation are all poorly understood. Several models of acute inflammation have revealed astrocyte subpopulations that go beyond a two‐activation state model, instead encompassing distinct functional subsets. However, how reactive astrocyte (RA) subsets evolve over time during chronic inflammatory disease or infection has been difficult to address. Here we use a prolific human pathogen, *Toxoplasma gondii*, that causes lifelong infection in the brain alongside a *Lcn2CreERT2* reporter mouse line to examine reactive astrocyte subsets during chronic neuroinflammation. Single‐cell RNA sequencing revealed diverse astrocyte populations including transcriptionally unique *Lcn2CreERT2*+ RAs which change over the course of infection in a subset‐dependent manner. In addition to an immune‐regulating *Lcn2CreERT2*+ astrocyte population enriched with gene transcripts encoding chemokines CCL5, CXCL9, CXCL10, and receptors CCR7 and IL7R, a specific subset of *Lcn2CreERT2*+ astrocytes highly expressed *transthyretin* (*Ttr*), a secreted carrier protein involved in glycolytic enzyme activation and potential vasculature regulation and angiogenesis. These findings provide novel information about the evolution and diversity of reactive astrocyte subtypes and functional signatures at different stages of infection, revealing an undocumented role for transthyretin‐expressing astrocytes in immune regulation at the central nervous system (CNS) vasculature.

## Main Points

- Toxoplasma infection results in distinct astrocyte subpopulations.
- Chronically labeled reactive astrocytes appear transcriptionally plastic and can regain acute inflammatory responding and naive homeostatic astrocytic gene patterns in the presence of ongoing neuroinflammation.
- Infection induces a *transthyretin* (*Ttr*)‐enriched chronic inflammatory glycolytic reactive astrocyte subpopulation found at the CNS vasculature.

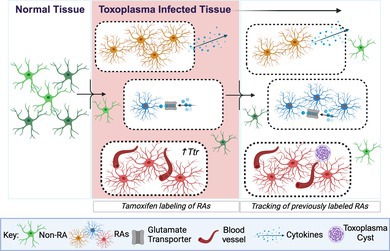

## 1 Introduction

Astrocytes are the most abundant glial cell type in the central nervous system (CNS) and have a central body with radiating branched or unbranched processes, allowing them to associate with numerous cells and tens of thousands of synapses (Verkhratsky et al. 2021). Critical homeostatic functions of astrocytes are fundamentally altered during brain inflammation and infection (Hidano et al. 2016; David et al. 2016). “Reactive astrogliosis” is the process by which astrocytes transform, in the presence of CNS insults, to “Reactive Astrocytes” (RAs) (Escartin et al. 2021), which encompass a heterogeneous population of cells within and across disease states (Sofroniew and Vinters 2010; Heppner et al. 2015; Anderson et al. 2016). Currently, an understanding of the dynamic nature of reactive astrocyte responses during a sustained immune response to chronic infection is lacking, including how RAs can potentially contribute to or prevent severe disease pathology (Fawcett and Asher 1999; Sofroniew 2015a; Liddelow and Barres 2017; Escartin et al. 2021; Fisher and Liddelow 2024).

Tools to identify and isolate reactive astrocytes are limited due to the diverse characteristics of these cells. Labeling for glial fibrillary acidic protein (GFAP) has been the most widely used tool for identification of reactive gliosis, as its expression increases significantly in many disease and injury models compared to the quiescent or baseline state (Abdelhak et al. 2018; Zheng et al. 2024). However, this approach has additional limitations: small subsets of “atypical” reactive astrocytes comprising roughly 6% of the total population have been identified in specific injury models such as mild TBI (Shandra et al. 2019). Additionally, CNS insults result in a mix of reactive and healthy astrocytes complicating the specific targeting of RAs (Escartin et al. 2021).

In the early 2010s, new reactive astrocyte markers were identified, including *Lipocalin‐2* (*Lcn2*) and *Complement‐3* (*C3*) (Escartin et al. 2021). Unlike GFAP, *Lcn2* shows no expression in the healthy adult brain but increases a few 100‐fold in models of systemic neuroinflammation and ischemic stroke (Zamanian et al. 2012). Upregulation of *Lcn2* in models of neuropathic pain, HIV‐related dementia, and Toxoplasma infection makes it particularly relevant for the study of reactive astrocytes in multiple disease and injury models (Ojeda‐Juárez et al. 2020; Agnew‐Svoboda et al. 2022).

While astrocyte reactivity has been observed in many contexts, more research is needed to understand how RAs might positively or negatively influence disease progression and resolution of inflammation. How distinct subsets of RAs may serve unique functions, similar to the diversity of resting state astrocytes, is also poorly understood (Escartin et al. 2021). Recent advances in single‐cell and spatial transcriptomics and proteomics provide unprecedented insight into astrocyte diversity (Anderson et al. 2016; Wheeler et al. 2020; Habib et al. 2020; Burda et al. 2022). These studies have shown that RAs likely serve diverse functions based on particular disease contexts and inflammatory conditions. For example, research on acute RA functions has uncovered the presence of wound healing and inflammatory regulating astrocytes during TBI and spinal cord injury (SCI) (Sofroniew 2015a; Todd et al. 2021; McCallum et al. 2024). The diverse nature of reactive astrocytes can be further observed in models of chronic inflammation and neurodegeneration, including Alzheimer's disease and Multiple Sclerosis, where subsets of RAs release various inflammatory mediators such as cytokines (*IL‐1B, IL‐17*, and *TNF‐a*) and cytotoxins (*Lcn2*) to induce severe inflammation (Zamanian et al. 2012; John Lin et al. 2017; Li et al. 2019; Hasel et al. 2021). While this diversity in putative function has been established for many disease models, the specific roles for different astrocyte subsets are poorly characterized. Additionally, further investigation is needed to determine whether astrocyte reactivity is reversible or progresses and evolves to support key immune system functions.

*Toxoplasma gondii* is a common neurotropic parasite that causes subclinical neuroinflammation due to lifelong cyst formation in neurons (Wohlfert et al. 2017). Astrocytes become reactive during Toxoplasma infection (Wilson and Hunter 2004; David et al. 2016). Survival of the host is dependent on the initiation of a classical type 1 immune response that results in recruitment of peripheral immune cells into the brain to prevent cell lysis, tissue damage, and encephalitis, caused by cyst reactivation. Astrocytes play multiple roles throughout this process, some neuroprotective such as inhibiting parasite replication via IFN‐γ STAT1 signaling pathways, while others potentially harmful such as downregulating glutamate transporter 1 (*GLT‐1*) expression and GABA signaling, contributing to elevated glutamate levels and excitotoxicity (Brooks et al. 2015; Hidano et al. 2016; David et al. 2016). There are multiple, and sometimes contradictory, roles for astrocytes during long term infection. Determining if all astrocytes are capable of all these functions simultaneously, or if subsets of cells have distinct phenotypes and roles, will help unravel the complexity of RAs in the immune response.

Astrocytes regulate the CNS vasculature by communicating with endothelial cells through their endfeet, emitting growth factors and extracellular matrix molecules to support blood vessels and encourage endothelial cell proliferation (Mathiisen et al. 2010; Walch et al. 2022; Puebla et al. 2022). Angiogenesis, the formation of new capillaries out of preexisting blood vessels, is crucial during development to support wound healing and supply oxygen‐rich blood to the brain. RAs upregulate angiogenesis‐associated genes following acute injury models such as stroke (Williamson et al. 2021). During *Toxoplasma* infection, there is vascular dysfunction and a reduction of angiogenesis in infected mice, ultimately promoting neuroinflammation (Estato et al. 2018).

Transthyretin (Ttr) is a tetrameric transport protein historically known for its capacity to carry thyroid hormone thyroxine and retinol‐binding protein (Alemi et al. 2016). It is synthesized in the liver and choroid plexus before being secreted into the bloodstream and cerebrospinal fluid (CSF) (Liz et al. 2020). In the CNS, Ttr plays a variety of roles associated with neuroprotection and repair in both acute and chronic brain encephalopathies (Gião et al. 2020). During acute injury, following cerebral ischemia, Ttr works to prevent neuronal cell death, edema, and inflammation (Santos et al. 2010) while during Alzheimer's disease, it can bind to Aβ to sequester and facilitate its clearance from the CNS (Alemi et al. 2016). Specifically, Ttr modulates glial energy metabolism via the cAMP‐dependent pathway (Zawiślak et al. 2017). Additionally, it is associated with CNS vasculature regulation, with several crucial genes involved in angiogenesis modulated by Ttr in human retinal microvascular endothelial cells during homeostatic and simulated diabetic retinopathy conditions (Nunes et al. 2013). Recent single‐cell RNA sequencing studies reported that *Ttr* was strongly upregulated in the medial vestibular nuclei after labyrinthectomy in two astrocytic clusters that were enriched for protective genes, such as metallothionein‐3 (*Mt3*), which is known to promote cell growth (Li, Wang, et al. 2023). Investigation into astrocyte‐derived *Ttr* in other environments can reveal new neuroprotective roles RAs play during disease.

In this study, analysis of RAs over the course of *Toxoplasma* infection demonstrates the emergence of multiple subpopulations that exhibit distinct transcriptional signatures. We found astrocyte populations with inflammatory or neuroprotective phenotypes, the proportion of which depended on the stage of infection. *Lcn2CreERT2*+ RAs are part of the neuroimmune response to Toxoplasma infection and exhibit a rich diversity that continually evolves from acute through chronic stages of disease similar to the differentiation, maturation and contraction of immune populations during a classical immune response. Finally, we identify a subpopulation of RAs highly enriched in *Ttr* expression and glycolytic transcripts in close proximity to blood vessels, suggesting a putative novel mechanism by which RAs might play neuroprotective roles during infection and disease through modulation of the cerebrovasculature.

## 2 Methods

### 2.1 Mice

All research was conducted in accordance with the Animal Welfare Act, and all efforts were made to minimize suffering. All protocols were approved by the Institutional Animal Care and Use Committee (IACUC) of the University of California, Riverside. Female wild type (WT) *C57BL/6J* mice were obtained from Jackson Laboratories and were maintained in a pathogen‐free environment in accordance with IACUC protocols at the University of California, Riverside. All experimental mice were used at 6‐ to 8‐week‐old age. The transgenic mouse line containing a tamoxifen‐dependent Cre driven by the *Lcn2* promotor was created by our labs as previously described (Agnew‐Svoboda et al. 2022). *Lcn2CreERT2* mice were maintained in the *C57BL/6J* background and crossed into the *Ai9* TdTomato reporter line available from JAX for experiments.

### 2.2 *Toxoplasma gondii* infections

To induce chronic *T. gondii* infection, mice were infected interperitoneally (IP) with the cyst‐forming Me49 Type II strain. Parasites were maintained in vivo by passaging alternatively between *Swiss Webster* (*SW*) and *CBA* mice (JAX). Following at least 3 weeks post‐infection, brains from *CBA* mice were harvested, homogenized in 3 mL sterile 1X phosphate buffered saline (PBS) through needle passing, and cysts were counted using a 30 μL aliquot and a light microscope (average cyst burden/brain ~3000). Cyst homogenate was diluted in sterile 1X PBS and 10 Me49 cysts were injected in experimental mice via 200 μL IP injections. This amount mimics physiological *T. gondii* infection that initiates in the gut of the host and travels through the bloodstream, eventually entering the brain to form neuronal cysts. Mice were monitored closely after infection to watch for any adverse effects.

### 2.3 Lcn2CreERT2;Ai9 Transgenic Reporter Mice

All mice were backcrossed over 10+ generations to the *C57BL/6J* strain to achieve a congenic background. Experimental mice were bred in‐house by crossing heterozygous transgenic lines expressing Cre recombinase under the specific promoter (*Lcn2CreERT2*) with a homozygous fluorescent reporter line *Rosa26 lsl‐tdTomato* (*Ai9*) to yield heterozygous experimental mice. *Lcn2CreERT2* transgenic mice were genotyped via PCR utilizing Transnetyx prior to and following breeding with reporter mice to verify transgene positivity.

### 2.4 Tamoxifen Induction and Timeline of Lcn2CreERT2 Promotor in Experimental Mice

To selectively activate reporter fluorescence of *Lcn2CreERT2*+ cells at chosen time points, mice were IP administered 150 μg/g of tamoxifen (Millipore Sigma, T5648) diluted in corn oil at the appropriate time points prior to the respective brain collections. Mice were then scarified via CO_2_ inhalation followed by decapitation according to IACUC guidelines. Astrocytes become reactive 5 days after initial infection (Wilson and Hunter 2004), therefore to label cells during acute infection, IP tamoxifen (TAM) injections were started at 5 days post‐infection (dpi) and given every other day until collection at 14 dpi (Figure 5b). For chronic infection, *Lcn2CreERT2*+ RA tdTomato red fluorescent protein expression was activated with TAM treatments starting on 14 dpi and given three times a week (MWF) until 42 dpi. To investigate how labeled *Lcn2CreERT2*+ RAs may change over time following long term infection, RAs were labeled from Day 14 to 42 dpi, tamoxifen was withdrawn, and the phenotype of the labeled *Lcn2CreERT2*+ RAs were analyzed after two additional weeks of infection (TAM‐rested). This timeline was chosen to label a wide range of astrocytes throughout the chronic state of *T. gondii* infection to determine specific potential chronic RAs in addition to any lasting changes.

### 2.5 Flow Cytometry

**FIGURE 1:**
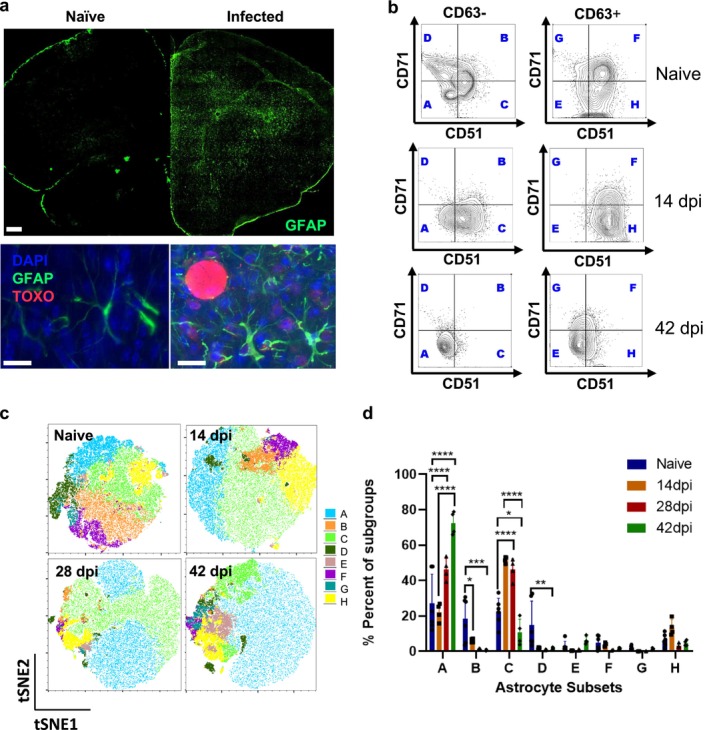
Diverse astrocyte subsets emerge over the course of *Toxoplasma gondii* infection. (a) GFAP expression (upper panels) in the naïve and chronically infected mouse brain at 42 days post infection (dpi). Images are stitched from XYZ. Images of astrocyte morphology and parasites (lower panels) assessed by DAPI (blue), anti‐GFAP (green) and anti‐Toxo (red) in coronal prefrontal cortex sections. (b) Gating strategy used to identify 8 A‐H astrocyte subpopulations based on expression of proteins CD63, CD51, and CD71 at each time point. (c) tSNE plots of all A‐H astrocyte populations as analyzed by flow cytometry over the course of infection. (d) Percentages of astrocyte subsets over the course of infection. *n* = 4 mice per infection time point and *n* = 6 for naïve. Statistics performed via two‐way ANOVA and pairwise comparison analysis. Statistical significance is labeled as: ^ns^ *p* > 0.05, *p ≤ 0.05, **p ≤ 0.01, ***p ≤ 0.001, ****p ≤ 0.0001. Scale bars upper (a) 500 μm, lower (a) 50 μm, coronal sections.

To isolate astrocytes, whole brains were collected from mice that were intracardially perfused with sterile 1x PBS at respective time points and placed in RPMI media (Gibco, 11875101) containing 1% fetal bovine serum (FBS) (Corning, MT35015CV) and 25 mM HEPES. Perfused brains were transferred to a chilled 60 mm Petri dish, dissociated with a blunt 18G needle, and treated with 4 mL of 0.25% trypsin. Brain suspension was digested at 37**°**C for 30 min in 50 mL conical tubes. During digestion, the mixture was inverted every 5 min. Trypsin digestion was stopped with the addition of 30 mL of RPMI containing 20% FBS and centrifuged at 1000 RCF (*g*) for 5 min at 4**°**C. Supernatant was decanted, and the pellet resuspended in 1% FBS RPMI to a total volume of 7 mL. The suspension was transferred to a 15 mL conical tube with 3 mL of 100% Percoll (Sigma Aldrich, P1644), gently mixed and underlaid with 1 mL of 70% Percoll made in 1% FBS RPMI. Samples were centrifuged at 2500 RCF (*g*) for 20 min at 4**°**C. Following density separation, cells were harvested from the interphase and washed in 1% FBS RPMI and resuspended in FACS buffer for flow cytometry staining. To avoid non‐specific binding, single cell suspensions were exposed to a purified rat anti‐mouse CD16/CD32 FC Block (BD Pharmingen, 553,142) at a 1:10 dilution in FACS Buffer (50 mg EDTA, 4 g BSA, 1 L 1XPBS) and incubated for 10 min. To detect astrocytes, cells were incubated for 30 min in intracellular anti‐GFAP at 1:50 in 0.3% saponin (ThermoFisher, 53‐9892‐80) following initial subgrouping of astrocytes via extracellular staining with anti‐CD51 (Biolegend, 104104), anti‐CD63 (ThermoFisher, 25‐0631‐82), and anti‐CD71 (BD Biosciences, 562858), each at a 1:50 dilution in FACS buffer and incubated for 30 min. Labeled cells were run on a NovoCyte Quanteon Flow Cytometer for acquisition only and on a MoFlo Astrios EQ Cell Sorter for cells sorting, both at the UCR School of Medicine Core Facility. GFAP and surface antigen positivity were determined via flow gating based on isotype controls specific to each protein. For flow analysis, subsets A‐H percentages in Figure 1, as well as whole astrocyte cell counts, were analyzed using FlowJo software. All statistical analyses were performed in GraphPad Prism (v9.0). Results of flow analysis for both *GFAP* levels and surface antigen expression, were compared using multiple comparisons two‐way ANOVA and pairwise comparison analysis. Statistical significance is labeled as: ns—*p* > 0.05, *p ≤ 0.05, **p ≤ 0.01, ***p ≤ 0.001, and ****p ≤ 0.0001.

### 2.6 Single Cell RNA Sequencing via 10X Genomics and Cell Sorting

To identify transcriptional astrocyte subsets, single cell RNA sequencing (scRNAseq) was performed on murine cells collected from whole brains. To obtain pure astrocyte samples, extracellular staining with anti‐astrocyte cell surface antigen‐2 (ACSA‐2) was performed (Miltenyi Biotec, 130‐117‐535). The viability stain 7‐aminoactinomycin (ThermoFischer, A1310) was added to FACS buffer to identify dead cells. ACSA‐2+ astrocytes were sorted and collected in a flow tube containing 10% FBS complete astrocyte RPMI with the Beckman Coulter MoFlo Astrios EQ Cell Sorter. For reporter experiments, sorting was performed on live cells using DAPI staining and tdTomato expression to collect live *Lcn2CreERT2*+ RAs for sequencing. A maximum of 16,000 astrocytes from each time point following the astrocyte cell suspension protocol was collected and pre‐loaded on a 10X Chromium chip and Chromium controller for barcoding. Single cell RNA sequencing steps from the 10X Genomics 3′ kit (Chromium Next GEM Single Cell 3′ Reagent Kits v3.1 PN‐1000128) were followed according to supplier instructions to extract RNA for sequencing.

### 2.7 Single Cell RNA Seq Purity Check and Data Analysis

RNA integrity throughout preparation was verified on the Agilent 2100 Bioanalyzer following cDNA amplification and library construction. When submitting samples to the 10X Genomics Core at UCSD, 1 billion reads per sample were requested to achieve a saturation level above 90%. Paired‐end sequencing experiments were performed on the UCSD Illumina NovaSeq 6000 and X Plus. After sequencing, the Cell Ranger program (v5.0.1) was utilized to align reads from the sequencing fastq data files to the mouse reference genome mm10 and generate gene‐barcode expression matrices. Mean reads and median genes per cell were reviewed for statistical relevance, and then aligned sequences were uploaded to R (v4.3.2) to generate plots and obtain gene statistics per cell and cluster. ScRNAseq analysis was performed using the *tidyverse* (v2.0) R package pipeline. Datasets for each set of collections were merged prior to performing quality control. Quality control was performed via filtering of each sequencing file with cut offs of RNA genes > 500, RNA counts (reads) > 500, and mitochondrial percentages < 20. Using Seurat (v5.0.1) data was log normalized, and the top 2000 most variable features were identified. The merged files were scaled, and linear dimensionality was performed to determine the number of principal components to use. Clustering using 20 principal components was performed, and a 0.1 resolution for BMNC/ASCA2 sorted data sets and 0.5 for *Lcn2CreERT2*+ sorted data sets were selected. Using 20 dimensions, the respective samples for each data set were anchored and integrated using canonical correlation analysis to find common cell populations. Scaling and linear dimensionality were performed using principal component analysis of the variable feature and non‐linear dimensional reduction in the form of a UMAP using 20 principal components. Clusters were identified by sets of known cell type marker genes (Figure S1d). Specific genes used to identify astrocytes were Aquaporin 4 (Aqp4), GFAP, glutamate synthetase (Glul), astrocytic calcium binding protein (S100b), GLAST (Slc1a3), glutamate transporter 1 gene (Slc1a2), alpha‐2 subunit gene for Na+/K+ ATPase (Atp1a2), and the gene that encodes glycolytic enzyme aldolase C (Aldoc). Astrocytes expressed at least 2 or more of these genes with a lack of other cell‐defining genes. Astrocytes were subsequently identified and clustered again by performing the same pipeline described above on only astrocyte clusters. Tools within the *tidyverse* pipeline also included *dplyr* (v1.1.4), *forcats* (v1.0.0), *lubridate* (v1.9.3), *purr* (1.0.2), *readr* (v2.1.4), *stringer* (v1.5.1), *tibble* (v.3.2.1), and *tidyr* (v 1.3.0). For scRNA‐seq analysis and data figures, we additionally used *ggplot2* (v3.4.4), *Seurat* (v5.0.1), *cowplot* (v1.1.1), RColorBrewer (v1.1–3), and *viridis* (v0.6.4). Tools within the *Bioconductor* (v3.17) project were additionally used including: *BiocManager* (v1.30.22), EnhancedVolcano (v1.20.0), *org.MM.eg.db* (v3.18.0), *annotationDbi* (v1.64.1), biomaRt (v2.58.0), monocle3 (v1.3.4), limma (v3.58.1), and *SeuratWrappers* (v0.3.2) for differentially expressed genes (DEG) volcano analysis, gene ontology (GO), and pseudo time analyses. For GO analyses, genes that had an average log2‐fold change above 0.5 were included. Volcano plots were made with a significant *p* value cut‐off value of 10e−32 and a fold change cut‐off of 1.5. All code for the file processing and analysis was performed through R via Seurat packages: All sequencing files were uploaded to the NCBI GEO database and GenBank (#GSE274474).

### 2.8 Tissue Preservation, Sectioning and Immunohistochemistry

Intracardial perfusions with 4% paraformaldehyde (PFA) in PBS was performed. Brains were fixed overnight at 4**°**C in 4% PFA and then cryoprotected with 30% sucrose solution. Cryoprotected brains were embedded in optimal cutting temperature (OCT) media and frozen over liquid nitrogen for later cryostat slicing. Tissue was cryosectioned at 10 μm thickness, fixed with 4% PFA, permeabilized with triton X‐100, and blocked with 5% donkey serum in PBS for 30 min at room temperature (RT). Tissue sections were incubated with the following selection of primary antibodies overnight at 4°C: anti‐GFAP (Rat anti‐GFAP, Invitrogen Cat# 2.2B10; at 2.5 μg/mL), anti‐Toxoplasma (Rabbit anti‐Toxo, Abcam Cat# ab138698; at 22 μg/mL), anti‐laminin (Rabbit anti‐Lam, EMD Millipore Cat# AB2034; at 5 μg/μL), and anti‐Transthyretin (Invitrogen, Cat# PA5‐20742; at 5 μg/mL) (Li et al. 2011; Talhada et al. 2020). Sections were washed with 1x PBS and incubated at room temperature for 1 h with secondary antibodies at 2 mg/mL: Goat anti‐chicken 647 (Invitrogen Cat# A21449), and Goat anti‐rat 488 (Life Technologies Cat# A11006). Both primary and secondary isotype controls for all antibodies were performed. Marker positivity was determined via microscopy. Negative and positive staining areas were quantified via experimenter‐blind counting and sizing. To determine if Ttr+ astrocytes colocalized with the vasculature sections were incubated with anti‐laminin, to identify the basement membrane of blood vessels along with anti‐Ttr, and anti‐GFAP. Colocalization of Ttr+; tdTomato+ and laminin was identified by overlap of staining at laminin positive sites. Ttr+; tdTomato+ were counted at laminin and non‐laminin areas as described below. To quantify Ttr+ and tdTomato+ RAs near *T. gondii* parasites and cysts tissues were incubated with anti‐Ttr, anti‐Toxo, and anti‐GFAP.

### 2.9 Image Analysis and Quantification

Images were taken with a BZ‐X800 all‐in‐one fluorescent microscope and a Leica DMi6000 inverted fluorescence microscope. Three brains, *n* = 3, were sectioned for each time point (acute, chronic, and TAM‐rested). For both antibody panels, each brain had three sections quantified from the cortical region with four regions of interest (ROIs) selected from each section. Random ROI selection was performed by taking four images per coronal brain slice using a set region pattern for cortex sections. A total of 36 regions were counted for each time point. Experimenter‐blinded counting of tdTomato+ astrocytes, Ttr, and Ttr proximity to laminin‐labeled blood vessels and cysts/parasites was performed using ImageJ. Statistics were run with GraphPad Prism using two‐way ANOVA. Tdtomato+ cells that touched laminin+ vessels (pink) or were within < 10 μm were counted as Ttr+. Additionally, Tdtomato+ cells in close proximity to cysts/parasites (< 10 μm) were counted and statistics were run in Prism using a two‐way ANOVA. Confocal imaging was performed using the same brain sections as described above, using an Olympus BX61 spinning disk confocal microscope equipped with a ×4, ×10, ×20, ×40 (oil) and ×60 (oil) objectives. Z‐stacks were defined by the upper and lower limits of the stack based on the morphology of tdTomato+ astrocyte bodies, with image slices taken every 1 μm at ×40 magnification. Z‐stacks were obtained from three independent biological replicates. Images were acquired using SlideBook 6 software. Image analysis was performed in Fiji to calculate the Manders' colocalization coefficient, comparing reactive astrocytes located in vascular versus nonvascular regions. Statistical analysis was performed in GraphPad Prism using two‐way ANOVA. Orthogonal views (*XZ* and *YZ* planes) of representative z‐stacks were generated using Imaris software to visualize the three‐dimensional spatial organization of TTR and tdTomato signals.

### 2.10 Statistics and Reproducibility

Independent biological replicates (mice) ranged from *n* = 3 to 6 for all individual experiments in this study and are indicated in each figure legend. Flow cytometry experiments include a minimum of four mice per time point and condition. Single‐cell RNA sequencing experiments have a minimum of six mice per time point and condition. Finally, immunohistochemistry experiments are from a minimum of three mice per time point, with 12 ROIs selected from each for quantification. All statistical tests used are listed in each figure legend. No data were excluded from the analyses.

## 3 Results

### 3.1 Diverse Astrocyte Subsets Emerge Over the Course of *T. gondii* Infection

Upregulation of GFAP, the production of cytokines and chemokines, and a capacity to kill parasites are responses of astrocytes following Toxoplasma infection (Halonen et al. 1998; Wilson and Hunter 2004; Hidano et al. 2016; David et al. 2016). Importantly, in chronically infected mice there is a clear upregulation of GFAP throughout the prefrontal cortex and around the lateral ventricles of the mouse brain, without any overt regional specificity (Figure 1a). However, GFAP labeling is homogeneous and therefore does not distinguish whether infection induces one population of reactive astrocytes or if subpopulations with distinct functional roles emerge. To address this question, we turned to previous comprehensive work identifying three transmembrane proteins, CD71, CD51, and CD63, that define subgroups of astrocytes (John Lin et al. 2017). In their study, Lin and colleagues purified Aldh1l1‐GFP+ astrocytes and sorted them based on the presence or absence of these markers. These subclasses of astrocytes were then phenotyped by flow cytometry, bulk RNA sequencing, and functional assays, focusing on subsets A‐E (John Lin et al. 2017; Table S1). Notably, subset A was found to demonstrate the highest migratory potential while subset C was found to be proliferative and important for synapse formation and function, revealing functionally diverse astrocyte subpopulations based solely on the presence or absence of these surface markers.

To determine whether a similar range of astrocyte heterogeneity is present during infection, we analyzed astrocytes by flow cytometry at 14‐, 28‐, and 42‐days post infection (dpi) (Figure 1b). These time points represent periods in which acute peripheral inflammation is migrating to the brain (14 dpi), followed by early and late (28‐ to 42‐ dpi) chronic CNS infection (Dubey 2014). In *C57BL/6* mice, infection and inflammation is progressive with increasing accumulation of parasites, T cells, activated microglia, and macrophages over time (Bergersen et al. 2021). In agreement with the previous work, astrocyte populations in uninfected mice were dominated by subsets A, B, C, and D (Figure 1b,c). During acute infection and early inflammation in the brain, there was a notable increase in subset C, a proliferative astrocyte population associated with synapse formation (Figure 1d) (John Lin et al. 2017). In contrast, late cortical developing astrocyte subsets B and D, representing approximately 20% of the naïve population, declined significantly after infection and were largely absent by early chronic infection (28 dpi). Subset C remained a significant proportion of astrocytes at 28 dpi, alongside an increase in subset A, known for its migratory capacity during early development. At the chronic stage of infection (42 dpi), subset A increased significantly, with a corresponding decrease in subset C. In addition to identifying previously described subsets primarily characterized by lack of CD63 expression (A‐E), Toxoplasma infection also induced small novel populations of astrocytes that upregulated CD63 and expressed CD51, CD71, or both (Figure 1; novel subsets F–H). These data reveal that infection induces significant changes in the diversity of astrocyte populations and that the relative proportions of these astrocyte subpopulations continue to change over the course of infection.

### 3.2 Transcriptome Gene Signatures Define Eight Astrocyte Subsets During *T. gondii* Infection

The ability to use surface proteins to distinguish astrocyte subsets has technical advantages (John Lin et al. 2017). However, it categorizes all cells based on three proteins, which likely underestimates the diversity of reactive astrocytes. Furthermore, it cannot be determined which of these populations are responsible for the multiple functions ascribed to RAs during infection. Therefore, to further investigate the distinctive functional components of astrocyte subpopulations during infection, cells were collected from naïve brains, acute (14 dpi), early chronic (28 dpi), and chronic (42 dpi) infected mice, and single cell RNA sequencing (scRNA‐seq) was performed to assess the transcriptional diversity of astrocytes following infection. To help identify astrocytes, unsorted brain mononuclear cells (BMNCs) and astrocyte cell surface antigen‐2 (ACSA‐2) sorted cells were compared (Figure S1a). Sequenced files were aggregated to compare datasets following quality control (see Section 2 and Figure S1b,c).

**FIGURE 2:**
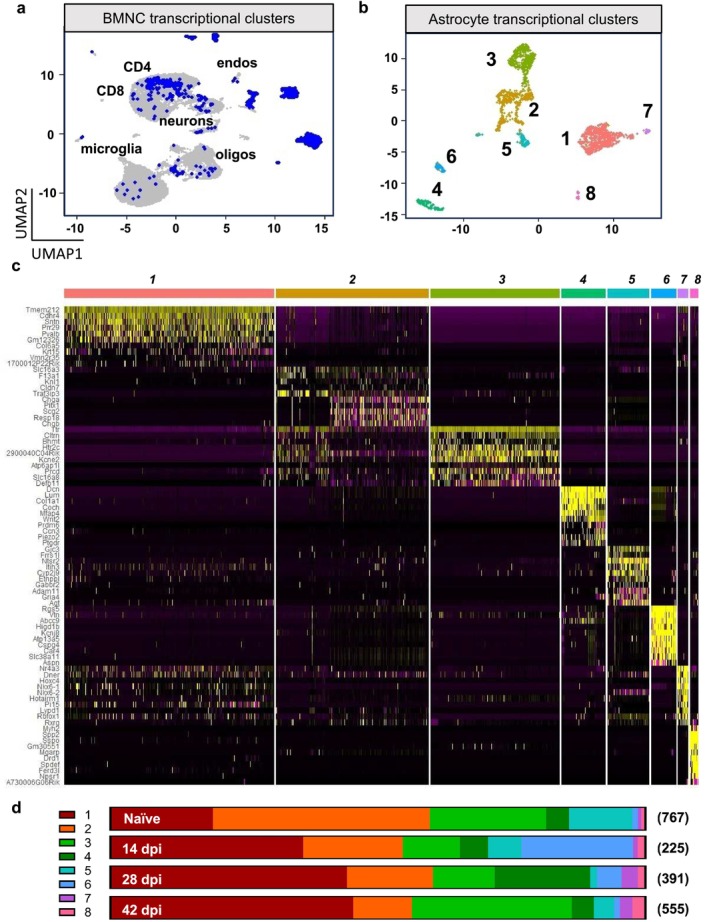
Transcriptomic gene signatures define eight astrocyte subsets during *T. gondii* infection. (a) Aggregated UMAP plot of both collection processes (BMNC and ACSA‐2 sorted) with all time points merged (naïve, acute 14 dpi, early chronic 28 dpi, and chronic 42 dpi). Astrocytes are highlighted in blue and major cell types labeled and identified as described in methods and Figure S1. (b) Re‐clustered UMAP plot of astrocytes identifies eight transcriptionally defined subpopulations (clusters 1–8). (c) Heatmap of the top 10 expressed genes in each cluster. (d) Stacked bar plots of the percentage of each cluster at each time point over the course of infection. *n* = 6 mice per time point. The number of astrocytes at each time point are indicated in brackets. (Additional heatmaps of the top 50 DEG by time point and cluster are provided in Figure S8).

Cell lineages could be determined from the expression of cell‐specific genes in the unsorted BMNC collection. Thus, infiltrating CD4+ and CD8+ T cells could be found by cluster expression of CD4 and CD8, while CNS resident endothelial cells, oligodendrocytes, microglia, and neurons were ascribed to clusters expressing respective cell‐specific genes listed in the heat map (Figure S1d). There were at least two clusters of cells with gene signatures that could be assigned to both microglia and astrocytes, highlighting the potential functional overlap of these cells and limitations of using one gene as an identifier (Figure 2a). A filter consisting of a list of astrocytic genes (see Section 2) was applied to all cells to identify astrocytes, and re‐clustering on these cells was performed. After initial re‐clustering, ~2000 pure astrocytes remained, which segregated into eight distinct clusters (Figures S1e and 2b).

These eight transcriptionally defined clusters were distinct from the subsets classified by the expression of CD63, CD71, and CD51 proteins (Figure 1). However, re‐clustering of astrocytes based on the genes encoding these proteins (CD71:*Tfrc*; CD51:*Itgav*; and CD63:*CD63*) revealed similar trends between gene‐ and protein‐defined subsets (Figures 1 and S2). Notably, subset A remained the largest population and increased over the course of infection, consistent with the flow cytometry experiments (Figures 1 and S2a,b). Populations C, E, and H were also detected at the transcriptional level and showed similar increases following infection as observed in the protein‐defined subsets (Figure S2a,b). To identify putative functions for astrocytes found particularly during chronic infection, subsets A and E were further analyzed for differentially expressed genes (DEGs) at 42 dpi. Enrichment of genes associated with the immune response including *Il1b*, transforming growth factor beta‐induced (*Tgfbi*), and immunoglobulin formation and potential antigen binding such as *Igkc* and *Ighg2c* were specific to subset A (Figure S2c). Recent studies suggest a complex role for astrocytes during the neuroimmune response, including the expression of aberrant immunoglobulins (Capuz et al. 2023). Gene ontology analysis revealed enrichment of pathways involved in high energy expenditure such as ATP and mitochondrial electron transport (Figure S2d). In contrast, subset E was defined by the upregulation of complement c1qa chain (*C1qA*), transthyretin (*Ttr*), and ectonucleotide pyrophosphatase/phosphodiesterase 2 (*Enpp2*) (Figure S2e). Gene ontology analysis revealed enrichment of pathways involved in synaptic pruning and regulation of calcium activity (Figure S3f). Although these data support the existence of the A‐H subsets, when analyzed across all time points following infection, astrocytes clustered into eight unbiased subsets not defined by CD71/*Tfrc*, CD51/*Itgav*, or *Cd63* expression. Therefore, to determine the effect of Toxoplasma infection on astrocyte population and function, we proceeded to analyze these eight unbiased subsets for gene expression and how these populations change over the course of infection (Figure 2b).

A heat map of the top 10 genes in each cluster highlighted non‐overlapping and distinct signatures of each subset (Figure 2c). For example, transporter *Slc16a3* gene, which encodes monocarboxylate transporter 4, important for lactate and known to be expressed by astrocytes in the CNS (Rafiki et al. 2003), was among the top genes expressed in Cluster 2. Cluster 1 astrocytes were enriched for *Tmem212* encoding transmembrane protein 212, cadherin gene *Cdhr4* important for cell adhesion and associated with cognitive ability (Goriounova and Mansvelder 2019), and parvalbumin encoding gene *Pvalb*. Parvalbumin is a calcium binding protein upregulated in reactive astrocytes and other glial cells following brain injury (Lichvarova et al. 2020). Cluster 3 showed enrichment of *prostaglandin synthetase* (*Ptgds*), a gene with multiple roles during inflammation and implicated in neuroprotective astrocytic functions (Choi et al. 2019). Enrichment of *decorin* (*Dcn*), an antagonist of TGF‐β (Schneider et al. 2021), suggests a further immune responsive astrocyte population in Cluster 4. Although all clusters had some expression of glutamate transporters, Cluster 5 was particularly enriched with 94% of cells in Cluster 5 expressing *Slc1a2* with an average log fold change of 5.624 and 90% expressing S*lc1a3* with an average log fold change of 5.096, genes encoding for the glutamate transporters Glt‐1 and GLAST, respectively. Thus, all eight clusters were enriched for genes known to be expressed by astrocytes.

To determine if Toxoplasma induced changes in the prevalence of specific astrocyte clusters over the course of infection, we mapped the proportion of each cluster over time (Figure 2d). Clusters 1, 7, and 8 expanded after infection, while Cluster 2 contracted. Cluster 2 continued to decline at each time point, from 40% in the naïve brain to 11% at 42 dpi, with a concomitant increase in Cluster 1 from 19% to 45% of the total population following infection. Cluster 6 expanded specifically during acute infection, making up 21% of astrocytes at 14 dpi, but by Day 42 was below background levels (Figure 2d). In contrast, Cluster 4 expanded only during early chronic infection, making up 18% of the astrocyte population at 28 dpi, but was otherwise very similar in size to naïve at 14 and 42 dpi. Cluster 3 was present at all stages but contracted following infection, until recovering at the late chronic stage to 30% of all astrocytes. Cluster 5 represented 12% of astrocytes in the uninfected brain but significantly dwindled to only 3% in the early chronically infected brain. The smallest populations, Clusters 7 and 8, were highest in the early‐ and late‐chronic time points, respectively. However, they never reached more than 3% of all astrocytes at any stage. Clusters 1 through 6 were identified as strong infection‐responsive clusters as their populations dramatically increased or decreased following infection (Figure 2d).

Overall, these data demonstrate that astrocytes undergo infection‐dependent transcriptomic changes resulting in dramatic shifts in distinct astrocyte subpopulations over the course of infection. Enrichment of populations of astrocytes that upregulate inflammatory‐responsive genes such as *Pvalb and Slc16a3* was observed, while astrocyte populations enriched for homeostatic glutamate transporter genes, such as *Slc1a2* and *Slc1a3*, decreased over the course of infection. The temporal course of astrocyte clusters indicates transient short‐term responding populations as well as expanding subsets over time, suggesting specific acute, early chronic, and chronic patterns of astrocyte heterogeneity during Toxoplasma infection.

### 3.3 Infection‐Induced Astrocytes Exhibit Distinct Gene Profile Populations

**FIGURE 3:**
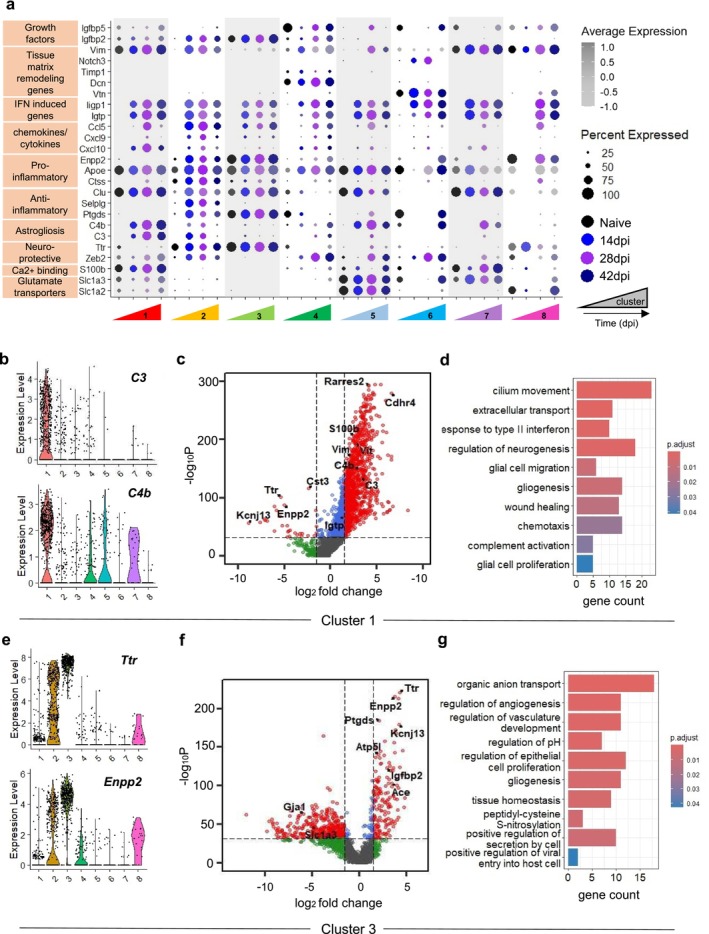
Infection‐induced astrocytes exhibit distinct gene profile populations. (a) Dot plot displaying the average expression levels of marker genes identified in each cluster. Each dot color corresponds to a time point: Naïve (black), acute 14 dpi (blue), early chronic 28 dpi (purple), and chronic 42 dpi (dark blue). The size of each dot indicates the percentage of cells within a cluster that express each gene, while the color intensity represents the average expression level of each gene across all cells within a cluster, with the darkest shade indicating the highest expression level. Triangles on the *x*‐axis represent increasing infection over time for each cluster. (b) Violin plots of strongly expressed genes in Cluster 1, *C3* and *C4b*. (c) Volcano plot of all genes with key upregulated genes *S100b*, *C4b*, *C3*, *Vim* and key downregulated genes *Ttr* and *Enpp2* highlighted. Red dots represent significant log10 *p* values and log 2‐fold changes, green indicates significant log 2‐fold change only, blue denotes significant *p* values only, while gray is not significant. (d) Gene ontology showing enriched biological pathways and the associated cell counts for Cluster 1. (e) Violin plots of strongly expressed genes in Cluster 3, *Ttr* and *Enpp2*. (f) Volcano plot of all genes revealed key upregulated genes *Ttr*, *Enpp2*, *Ptgds* and key downregulated genes *Slc1a3* and *Gja1* in Cluster 3. (g) Gene ontology showing enriched biological pathways and the associated cell counts for Cluster 3.

To understand how astrocyte populations relate to known astrocyte functions during Toxoplasma infection, we focused on genes involved in inflammatory and anti‐parasitic processes and determined whether they were enriched in all clusters or only in specific subsets. Levels of expression for each gene within each cluster compared to all astrocytes were visualized in a dot plot (Figure 3a). A primary role for astrocytes during infection is the production of chemokines to recruit protective immune cells from the periphery (Sofroniew 2015b; Still et al. 2020; Orchanian et al. 2024). The three most upregulated chemokines during infection were *Ccl5*, *Cxcl9*, and *Cxcl10*. Transcripts for these genes were only present during infection and confined mainly to Clusters 1–4. Specifically, *Cxcl10* was present and upregulated in over 75% of Cluster 1 astrocytes during early chronic infection, yet decreased to approximately 50% during chronic infection, suggesting a reduction in inflammation (or need) during chronic infection. A population‐defining gene enriched in Cluster 2 was *Ccl5* encoding chemokine CCL5 (Figure 3a). CCL5 is a chemoattractant for T cells, which are required to control parasite replication. *Ccl5* was present in 100% of Cluster 2 astrocytes during acute and early chronic infection but decreased to approximately 75% during late chronic infection in a pattern similar to *Cxcl10*. Although other chemokines were found throughout all astrocyte subpopulations, Cluster 2 specifically expressed *Ccl5* during chronic infection. Glutamate transporter genes (*Slc1a2*, *Slc1a3*) were significantly enriched in Cluster 5. This enrichment decreased over the course of infection (Figure S3g) along with a reduction in the proportion of astrocytes in Cluster 5 (Figure 2d), suggesting a possible loss of astrocyte glutamate uptake. This is in agreement with our previously published work identifying downregulation of Glt‐1 and upregulation of extracellular glutamate concentrations following infection (David et al. 2016). Loss of Cluster 5 after infection could indicate a reassignment of Cluster 5 astrocytes into other clusters. In support of this, low expression of glutamate transporter genes was apparent in the inflammatory‐responsive astrocyte Clusters 1 and 2 (Figure 3a). Additional pro‐inflammatory and neuroprotective genes implicated in other disease states (*Enpp2*, *Ttr*, *Ptdgs*) were expressed in Cluster 3 at both early and late chronic infection time points.

To identify the potential roles of astrocytes during chronic infection, we focused on Clusters 1 and 3 which, when combined, made up over half of all astrocytes at 42 dpi and showed the greatest sustained change from naïve to chronic infection (Figure 2d). In addition to upregulation of *Cxcl10*, Cluster 1 had elevated complement protein 3 (*C3*) transcripts, a proinflammatory and known reactive astrocyte marker (Phares et al. 2013; Doron et al. 2019; Liang et al. 2023). Increased cytokine production supports the idea that this astrocyte subgroup is an important mediator of the inflammatory response during Toxoplasma infection (Figure 3b,c). *Complement 3* is known to play a crucial role in neurodevelopmental processes such as neuronal migration and synaptic pruning (Gorelik et al. 2017), and is released from astrocytes during Toxoplasma infection, AD, and MS (Carrillo et al. 2023; Stym‐Popper et al. 2023; Liddelow et al. 2017). The precursor of C3, convertase, *C4b*, was also enriched in Cluster 1 and was also present in Clusters 4, 5, and 7 (Figure 3a,c). Gene ontology (GO) analysis of Cluster 1 revealed pathways enriched in glial cell migration and proliferation, including the terms “cilium movement” and “microtubule‐based movement” (Figure 3d). We also observed enhancement of pathways involved in the immune response, specifically “response to type II interferon.” Type II interferon, IFN‐γ, signals through the *JAK/STAT1* signaling pathway and is the essential cytokine required to prevent parasite replication in astrocytes and is required for host survival following infection (Hidano et al. 2016) (Figure 3d). In line with this immune‐centric phenotype, Cluster 1 showed loss of potential neuroprotective genes such as *Ptgds*, *Ttr*, and *Enpp2*, all genes that were instead heavily upregulated in Cluster 3. This shift in gene expression across clusters further supports reactive astrocyte heterogeneity and that certain subsets emerge to perform specific functions during infection. Overall, the data suggest that Cluster 1 is an immune responsive astrocyte population directed toward a type II interferon activated mechanism.

The second major cluster found to change over the course of infection was Cluster 3, which made up 20% and 30% of astrocytes at the naïve and late chronic time points, respectively, but only comprised about 10% of astrocytes at 14 and 28 dpi (Figure 2d). Genes upregulated in Cluster 3 included *Ttr*, *Enpp2*, and *Ptgds* (Figure 3e,f). *Ttr* stimulates expression of glycolytic enzymes in astrocytes resulting in increased production of ATP, suggesting an astrocyte responding subset with increased metabolic activity (Zawiślak et al. 2017; Morel et al. 2019). *Enpp2* encodes *autotaxin* (*Atx*), which helps convert lysophospholipids into a G‐protein coupled receptor ligand, *lysophosphatidic acid* (*LPA*). Studies using neuronal‐astrocyte mixed cultures revealed that treatment with *LPA* results in elevated neuronal intracellular calcium levels and a decrease in astrocytic glutamate uptake, a key pathology seen in chronic Toxoplasmosis (Ramesh et al. 2018; David et al. 2016). In addition, GO term analysis found enhancement of pathways involved in “regulation of pH,” “positive regulation of secretion,” and “regulation of vascular development and angiogenesis” (Figure 3g). Overall, Cluster 3 appears to be a putative energy expending astrocyte population with a potential contribution to vasculature regulation.

Our data suggest that astrocytes have multiple roles in immune regulation, vascular regulation, and possible neuroprotection during Toxoplasma‐induced neuroinflammation and that there are distinct populations responsible for each of these functions. In addition, new highly upregulated genes found in these subsets suggest previously unidentified roles for reactive astrocytes.

### 3.4 Astrocyte Populations Have Diverse Gene Expression Signatures Throughout Infection

Further analysis of the differentially expressed genes (DEGs) and cluster‐defining genes on the remaining astrocyte subpopulations revealed transcriptional signatures suggesting distinct cellular functions. All analyses were performed at the 42 dpi time point. Astrocyte Clusters 2 and 5, which made up the majority of naive astrocytes but were largely lost following infection, were enriched for NF‐κB target gene *cathepsin S* (*Ctss*) and Glt‐1 encoding gene, *Slc1a2*, respectively (Figure S3a,b,g,h). *Ctss* upregulates *Jak2ca*, in the JAK2‐STAT3 signaling cascade in astrocytes during Parkinson's disease, increasing proteolytic capacity in astrocytes and ultimately activating a beneficial proteostasis function in RAs (Abjean et al. 2023). In alignment with those findings, in Cluster 2 we found upregulation of pathways associated with extracellular matrix organization and wound healing, processes known to take place during chronic infection (McGovern et al. 2021; Nance et al. 2012) (Figure S3c). Expression of *Slc1a2* was also significantly downregulated in Clusters 1 and 8 relative to naïve uninfected mice (Figure 3a), even though these populations expanded after infection. Overall, these findings demonstrate that although subpopulations of reactive astrocytes are heterogeneous in their expression of genes encoding glutamate transporters, there is an overall loss of glutamate transporter gene expression following infection. Cluster 5 was also enriched in pathways involved in gliogenesis, glial cell differentiation and development, and regulation of synapse organization, all of which are well‐defined astrocytic homeostatic functions (Figure S3i). Clusters 4 and 7, which were prominent during the early chronic time point, had upregulation of *Decorin* (*Dcn*) and *Hox* genes, respectively (Figure S3d,e,m). Enhancement of pathways involved in immune regulation, astrocyte activation and development were seen in Cluster 4 (Figure S3f) and Cluster 7. Enriched pathways included extracellular transport and gliogenesis (Figure S3n). Cluster 6, which expanded only during acute infection, upregulated expression of *Vitronectin* (*Vtn*), known for its involvement in tissue remodeling (Ruzha et al. 2022; Figure S3j,k). Further corroborating these functions, Cluster 6 enrichment pathways included wound healing, cell migration, and extracellular matrix reorganization (Figure S3l). The smallest cluster, Cluster 8, was most prominent during the chronic 42 dpi time point and was characterized by the upregulation of *secreted phosphoprotein 2* (*Spp2*) (Figure S3o) and enriched pathways involved in neurogenesis, extracellular transport, and axon guidance (Figure S3p).

### 3.5 Dynamic Populations of *Lcn2CreERT2;Ai9* Reporter Astrocytes Over the Course of Infection

**FIGURE 4:**
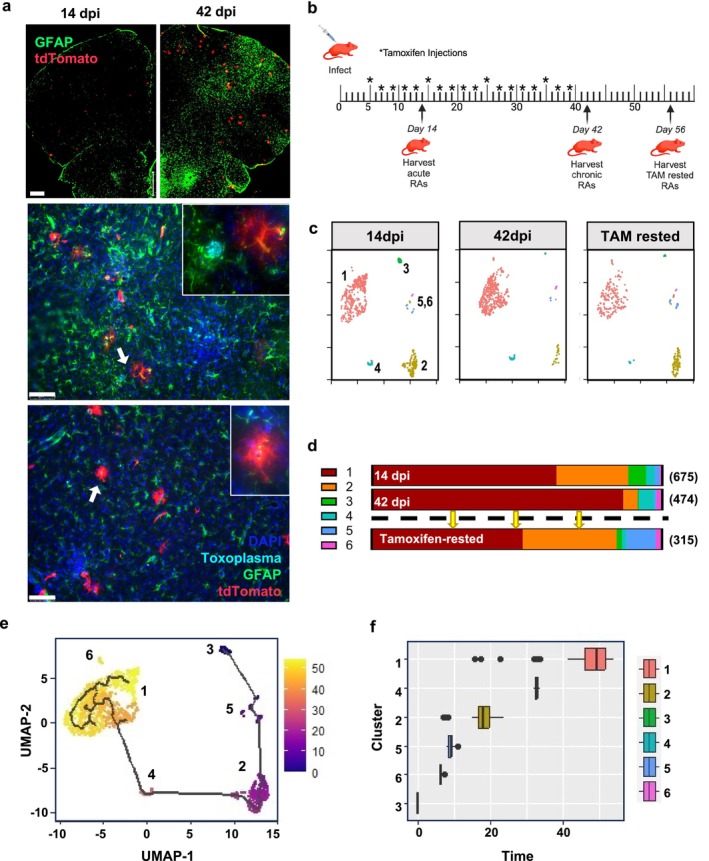
Unique reactive astrocyte subsets defined by *Lcn2‐CreERT2;Ai9* reporter expression. (a) 12 μm Lcn2CreErt2;Ai9 coronal sections stained for GFAP (green) and tdTomato signal (red) in the cortex of 14‐dpi mice (left top) and 42‐dpi mice (top right). Overlay of red and green signal indicates *Lcn2‐CreERT2*‐expressing reactive astrocytes. Middle and lower panels show Lcn2CreErt2;Ai9 42‐dpi brain sections stained for DAPI (blue), anti‐GFAP (green), anti‐Toxo (turquoise), and tdTomato (red). (b) Experimental timeline of tamoxifen administration (arrows) for *Lcn2* ^*CreERT2*^;*Ai9* mice infected with *T. gondii* at Day 0 and tissue collected at various days post‐infection (dpi) (acute 14 dpi, chronic 42 dpi, tamoxifen‐rested time point). (c) UMAP of the 6 *Lcn2CreERT2*+ RA clusters at each time point 14 dpi, 42 dpi, and tamoxifen‐rested. (d) Stacked bar plots of each cluster at each time point. The respective number of astrocytes are indicated in the brackets per condition. (e) UMAP of the six *Lcn2CreERT2*+ clusters plotted with pseudo‐time analysis and corresponding box plots (f) showing progression and emergence over time of each astrocyte cluster. Scale bars top (a) 500 μm, middle and bottom (a) 75 μm. *n* = 6 per time point.

*Lipocalin 2* is consistently upregulated in astrocytes during inflammation following CNS insult, but minimally expressed in the healthy brain (Zamanian et al. 2012; Lee et al. 2015; Li, Xu, et al. 2023; Agnew‐Svoboda et al. 2022). *Lcn2* expression, although variable, was found in most astrocyte populations from bulk sequencing following infection but never in the uninfected brain, confirming its ability to identify subpopulations of reactive astrocytes (Zamanian et al. 2012). Therefore, in addition to sorting for astrocytes based on ACSA‐2, we crossed the *Lcn2CreERT2* transgenic mouse line with the fluorescent *Ai9* reporter to label and define a subset of reactive astrocytes during Toxoplasma infection, as described previously (Agnew‐Svoboda et al. 2022) (Figure 4a,b) (see Section 2). This system permits labeling and longitudinal tracking of a subset of *Lcn2*+ RAs to test if reactive astrogliosis is heterogeneous, progressive, resolves, or changes across specific disease time points.

Acute 14 dpi and chronic 42 dpi astrocytes collected from *Lcn2CreERT2*+ RAs were labeled and analyzed using the tamoxifen regimens described in Methods (Figure 4b). In addition, labeling of astrocytes was conducted up until 42 dpi, but tamoxifen administration was halted for 2 weeks prior to analysis at 56 dpi (Tamoxifen‐rested group). This allowed labeled RAs to be tracked over time to determine if astrocytes change phenotype between chronic versus late chronic time points. Single‐cell RNA sequencing was performed on tdTomato+ (Lcn2‐Cre expressing) cells, and following quality control (see Section 2), cells were determined to be either endothelial cells or astrocytes, which combined made up 90% of all sorted cells (Figure S4a–c) as we observed previously in other models of inflammation (Agnew‐Svoboda et al. 2022). A filter based on a list of astrocytic genes (see Section 2) was applied to all cells in the data to identify astrocytes, and re‐clustering on these cells was performed. After re‐clustering, ~1500 *Lcn2CreERT2*+ astrocytes remained, and six subpopulations were identified (Figure 4c,d).

We first assessed whether *Lcn2CreERT2*+ astrocyte populations changed over time by comparing the 56 dpi “tamoxifen‐rested” time point to acute (14 dpi) and chronic (42 dpi) time points (Figure 4c,d). Based on cluster representations, when tamoxifen was withdrawn, *Lcn2CreERT2*+ RAs were more similar to RAs at 14 dpi than to chronic 42 dpi RAs, suggesting a partial reversion of RAs back to an acute response state. Specifically, *Lcn2CreERT2*+ RA Cluster 1 expanded from 63.7% at 14 dpi to 86.5% at 42 dpi (Figure 2d). However, with no labeling of new RAs, the proportion of astrocytes in Cluster 1 was reduced to 52.1% 2 weeks later, with a concomitant increase in *Lcn2CreERT2*+ Cluster 2 from 5.1% at 42 dpi to 32.3%. Expansion of Cluster 2 was similar to the Cluster 2 proportion of RAs at 14 dpi (24.7%), suggesting a dynamic and plastic RA population that can change over the course of infection to support the immune response. Lastly, Cluster 5, enriched for glutamate transporter genes *Slc1a2* and *Slc1a3*, increased from 1.8% during acute infection and from only 0.84% at 42 dpi to 10.2% during the tamoxifen‐rested time point, suggesting partial recovery of glutamate transporter expression during prolonged chronic infection in this subpopulation of RAs. These data suggest that during chronic infection some astrocytes have the ability to revert to acute responding RAs and potentially restore neuroprotective and homeostatic functions.

Analysis of gene signatures associated with cell cycle and replication using pseudo‐time analysis was performed on the six *Lcn2CreERT2*+ RA astrocyte clusters to help determine if subsets of RAs are differentiating into other clusters. This analysis revealed early versus late differentiating populations (Figure 4e,f). Cluster 3 appeared as the oldest most fully differentiated population, while Cluster 1, with the highest pseudotime value, was the most recently emerging population. Expansion of Cluster 1 during chronic infection would support the concept that Cluster 1 is a recently differentiated RA population.

### 3.6 *Lcn2CreERT2* + RAs are Involved in Immune Regulation and Have a Re‐Emergence of Glutamate Transporter Gene Expression

**FIGURE 5:**
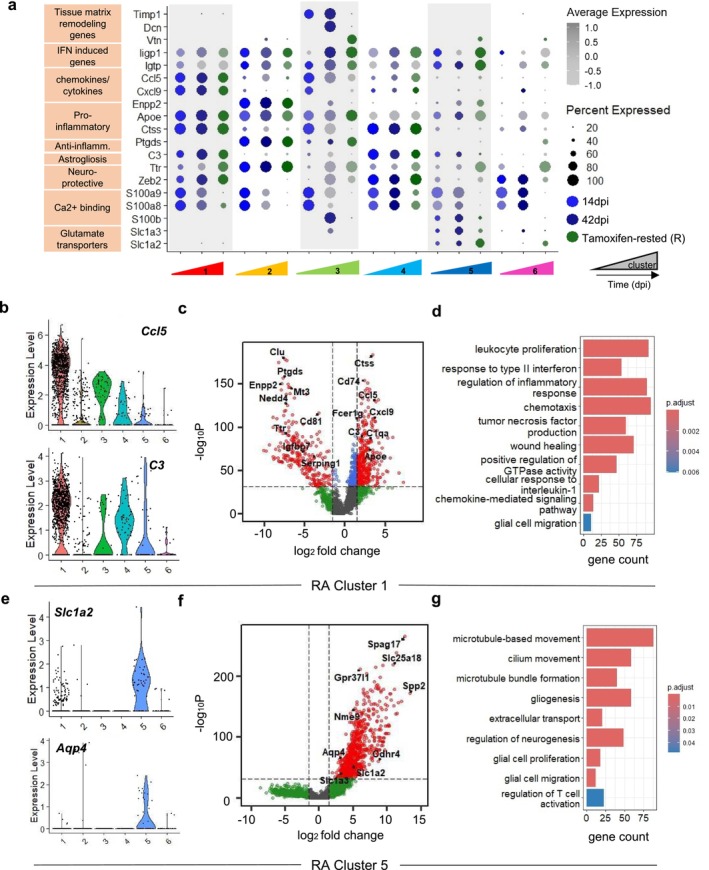
Lcn2CreERT2+ RAs are involved in immune regulation and have a re‐emergence of glutamate transporter gene expression. (a) Dot plot displaying the average expression of phenotypic genes identified in each cluster. Time points are color‐coded, with acute 14 dpi (blue), chronic 42 dpi (dark blue), and tamoxifen‐rested (green). Triangles on the *x*‐axis represent increasing infection over time for each cluster. (b) Violin plots of strongly expressed genes in Cluster 1, *C3* and *Ccl5*. (c) Volcano plot of all differentially regulated genes in Cluster 1. Red dots represent significant log10 *p* values and log 2‐fold changes, green indicates significant log 2‐fold change only, blue denotes significant *p* values only, and gray is not significant. (d) Gene ontology analysis of enriched biological pathways and the associated gene counts for Cluster 1. (e) Violin plot of a Cluster 5 defining genes, *Slc1a2 and Aqp4*. (f) Volcano plot of all differentially regulated genes in Cluster 5. (g) Gene ontology analysis of enriched biological pathways with corresponding gene counts for Cluster 5. (Additional heatmaps of top 50 DEG by time point and cluster are provided in Figure S9).

Heatmap analysis of the top 10 genes in each cluster highlighted the driving gene signatures in each subset and suggested functional differences between *Lcn2CreERT2*+ RA populations (Figure S4d). In an effort to correlate *Lcn2CreERT2*+ astrocyte clusters with function, we identified highly significant genes among the six clusters over the course of infection (Figure 5a). To identify potential roles of *Lcn2CreERT2*+ RAs during chronic infection, we focused on Cluster 1, which made up over 85% of all *Lcn2CreERT2*+ RAs at 42 dpi, and over 52% of all RAs at the tamoxifen‐rested time point; and Cluster 5, which represents a small component of RAs during acute and chronic infection but expanded to more than 10% of all RAs between 42 and 56 dpi.

Cluster 1 had enrichment of genes for immune regulation (Figures 5a–d and S4d). These included chemokine receptor 7 (*Ccr7*), which is activated by ligands CCL19 and CCL21 and is upregulated in RAs following intracerebral LPS (Gomez‐Nicola et al. 2010) and during chronic Toxoplasma infection (Ploix et al. 2011; Noor and Wilson 2012; McGovern et al. 2021); interleukin 7 receptor (*Il7r*), which when downregulated in astrocytes in EAE mice contributes to demyelination and progression of disease (Hamby et al. 2012); and inflammation mediator purinergic receptor gene *P2y14* (Lei et al. 2017). Proinflammatory chemokine genes *Ccl5* and *Cxcl9* were highly expressed by RAs at all time points in Cluster 1. Cluster 1 was also notable for the absence of genes *Ptgds* and *Enpp2*, which instead were enriched in Cluster 2 astrocytes. In addition to chemokine expression, Cluster 1 was distinguished by the high expression of *Complement 3* and *Ctss* (Figure 5a–c). Gene ontology analysis indicated upregulation of pathways associated with “regulation of inflammation” and “response to type II interferon” (Figure 5d). In contrast, Cluster 5 was enriched in glutamate transporter *Slc1a2* and *Aqp4*. Other astrocytic homeostatic genes such as *Slc1a3* were also specifically enriched in a Cluster 5 (Figure 5e,f). GO terms included “glial cell migration” and “gliogenesis” (Figure 5g). In addition, expansion of Cluster 5 after 2 weeks of tamoxifen withdrawal (Figure 4c), suggests the re‐emergence of a supportive astrocyte population that is more competent in glutamate transport, regulation of interstitial volume, and the glymphatic system.

Additional cluster analysis showed in Cluster 3 an upregulation of *matrix gla protein* (*Mgp*), significantly overexpressed in astrocytic tumors, and enrichment of GO pathways associated with immune regulation including “lymphocyte differentiation” and “regulation of T cell activation” (Mertsch et al. 2009; Figure S5a–c). Cluster 4 was defined by the expression of *Serpinb2*, known for regulation of the adaptive immune response, and enrichment of pathways that regulate various adaptive immune processes (Schroder et al. 2010; Figure S5d–f). Upregulation of *Nrxn3* and *Tubb3* were observed in Cluster 6, with enriched pathways in “gliogenesis” and “regulation of neurogenesis” (Figure S5g–i).

Taken together, these findings suggest that RAs defined by *Lcn2CreERT2*+ reporter expression are also subdivided into several heterogeneous subpopulations with distinct transcriptional signatures, suggesting specific functional roles for reactive astrocytes during acute and chronic *Toxoplasma* infection. In addition, based on fluctuations in cluster populations over the course of infection, *Lcn2CreERT2*+ RAs seem capable of regaining lost astrocyte housekeeping roles, such as the re‐emergence of *Slc1a2* expressing RAs to possibly recover glutamate transport function. Furthermore, the expansion of Cluster 5 from previously labeled astrocytes supports the dynamic nature of RAs during infection‐induced neuroinflammation.

### 3.7 A Subset of *Lcn2CreERT2* + Ttr‐Enriched RAs Are Enriched in Glycolytic Genes and Are Found at the Vasculature

**FIGURE 6:**
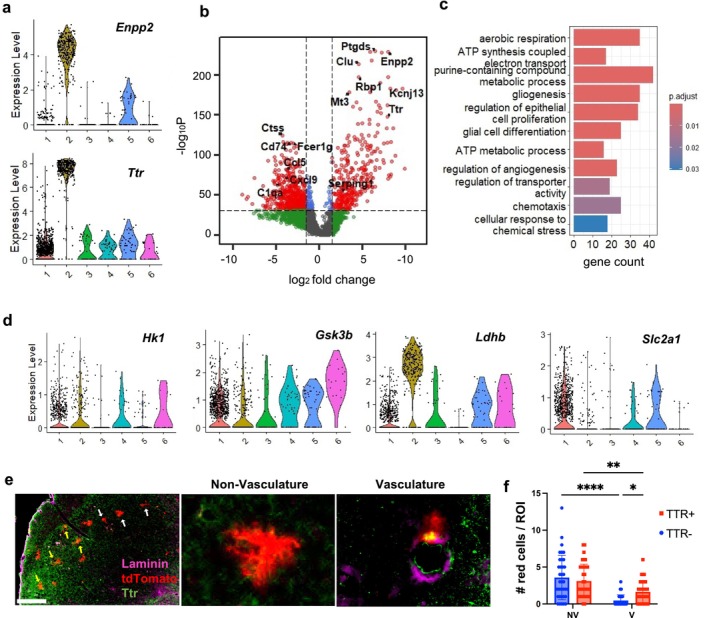
A subset of *Lcn2CreERT2*+ *Ttr*‐expressing reactive astrocytes are found at the CNS vasculature. (a) Violin plots of strongly expressed genes in Cluster 2, *Enpp2* and *Ttr*. (b) Volcano plot of all differentially expressed genes in Cluster 2. (c) Gene ontology analysis of enriched biological pathways. (d) Violin plots of glycolytic, lactate, glucose transporter, and metabolic genes (*Hk1*, *Slc2a1, Ldha, Ldhb, Gsk3a, Gsk3b, Pygb*, and *Gys1*) present during chronic infection in *Ttr* enriched RA cluster 2 (*also see* Figure S6a,b). (e) 12 μm cortical coronal sections near blood vessels stained for laminin (purple) to label the vasculature, transthyretin (green), and Lcn2Cre + RAs (red). *Yellow arrows indicate RAs colocalized with Ttr, *white arrows indicate RAs not colocalized with Ttr. (f) Quantification of percentages of Ttr‐labeled RAs near and away from laminin‐stained blood vessels performed at ×100 magnification. *N* = 3 mice per time point, three slices per brain, four regions per slice. Scale bar, 50 μm. *p* values: Comparison between TTR+ and TTR‐ at the vasculature *p < 0.05 (*p* = 0.0173); Comparison of TTR+ RAs at the vasculature versus not at the vasculature **p < 0.01 (0.0028); Comparison between TTR‐ at the vasculature or not at the vasculature ****p < 0.0001.

To identify potential roles of *Ttr*‐enriched *Lcn2CreERT2*+ RAs during chronic infection, we focused on Cluster 2, which was prevalent during acute infection, and expanded from 5% of RAs labeled at 42 dpi to more than 30% of RAs when tracked for a further 2 weeks (Figure 4c). Cluster 2 defining genes included *Ttr*, *Enpp2*, and *Ptdgs*, which was similar to what we observed for Cluster 3 in all astrocytes sorted by ACSA‐2 (Figures 3a,e,f and 6a,b). GO terms enriched in Cluster 2 at 42 dpi included those involved in angiogenesis and metabolic processes, indicating a potential role for *Lcn2CreERT2*+ reactive astrocytes in metabolism and vasculature regulation (Figure 6c).

Because *Ttr* has been shown to promote glycolysis (Zawiślak et al. 2017), we analyzed glycolytic and metabolic gene expression in *Ttr*‐enriched clusters from both reporter (Cluster 2) and non‐reporter (Cluster 3) scRNA‐seq datasets (Figures 6d and S6–S8). In both datasets, we observed increased expression of key glycolytic and metabolic genes following infection. The regulation of glycolysis depends on the availability of the substrate and enzymatic product concentrations and is controlled by three rate‐limiting enzymes: hexokinases (HKs), phosphofructokinase‐1 (PFK‐1), and pyruvate kinases (PKs) (Figure S6b). Hk1, which catalyzes the rate‐limiting steps of glycolysis, was expressed by the *T*tr‐enriched reporter dataset Cluster 2, and this expression increased over the course of infection (Figures 6d, S6 and S7). The presence of other glycolytic enzymes, including aldolase (*Aldoa*), *Gpi1*, *Pgk1*, *Eno1*, *Pkm*, and *Gapdh* further indicated a strong commitment to glycolysis in this cluster (Figures S6 and S7). Similarly, these enzymes were present in enriched *Ttr* Cluster 3 of the non‐reporter dataset (Figure S8). Additionally, we also observed upregulation of *Gsk3b* and *Pygb*, which are enzymes involved in glycogen metabolism (Zhang et al. 2021; Figures S7 and S8) Interestingly, both clusters showed slightly higher expression of *Ldhb* compared to *Ldha*, suggesting a preference for converting pyruvate to lactate and regeneration of NAD+ – a critical process for maintaining ATP production under anaerobic conditions (Figures 6d, S6 and S8) (Alberini et al. 2018). Consistent with this, glucose transporters (*Slc2a1, Slc2a3*) and monocarboxylic transporters 1 and 4 (*Slc16a1, Slc16a3*) appeared to have low expression in this cluster, suggesting that *Ttr*‐enriched astrocytes may limit the release of lactate for uptake by neurons due to diminished glucose transport capacity (Tao et al. 2022; Figures 6e, S6a, S7, and S8). Overall, Ttr‐expressing RAs may shift away from energy storage in favor of energy consumption through increased metabolism of glycogen stores as well as finding alternative ways to produce energy through the TCA cycle.

### 3.8 A Subset of *Lcn2CreERT2* + Ttr‐Expressing RAs Is Found at the CNS Vasculature

Transthyretin, a known transporter of vitamin A and thyroxine, has not been heavily studied in astrocytes aside from its ability to modulate glial energy metabolism (Zawiślak et al. 2017). Accumulating evidence highlights the diverse roles Ttr plays in the CNS, including a protective role during neuroinflammation with its ability to bind to Aβ and facilitate its clearance (Alemi et al. 2016). Ttr has also been implicated in the regulation of angiogenesis by modulating endothelial cells, yet this did not define any contribution from astrocytes (Nunes et al. 2013). The striking prevalence of *Ttr* expression in Cluster 2 (Figure 6a) warranted further investigation. To determine if *Ttr*‐expressing astrocytes were localized to the vasculature, we performed immunohistochemistry for Ttr and GFAP in brain sections from *Lcn2‐CreERT2;Ai9* mice at acute and chronic time points and used laminin to identify the basement membrane of blood vessels. We analyzed *Lcn2CreERT2*+ RAs both adjacent to (< 10 μm) and away from (> 10 μm) laminin‐stained blood vessels to identify Ttr+ RA expression patterns (Figure 6e,f). Characteristic astrocyte morphology together with colocalization of GFAP and tdTomato signal was used to identify *Lcn2CreERT2*+ expressing RAs (Figures S9 and S10). Using these parameters, approximately 50% of tdTomato signal was astrocytic, while the remainder appeared to be endothelial cells, corroborating both our *Lcn2CreERT2*+ scRNAseq data and previous findings (Figure S9b, Agnew‐Svoboda et al. 2022). Total cell counts revealed an increase in *Lcn2CreERT2*+ RAs at 42 dpi compared to acute infection as expected (Figure S9b). Notably, perivascular *Lcn2CreERT2*+ RAs were significantly more likely to be Ttr+ whereas approximately only half of *Lcn2CreERT2*+ RAs located away from blood vessels were Ttr+ (Figures 6e,f, S9, and S10). This supports the possibility that Ttr‐expressing astrocytes are involved in angiogenesis pathways (Figure 6h,i).

Lastly, we investigated whether *Lcn2CreERT2*+ RAs were associated with the presence of Toxoplasma cysts or were more randomly distributed within the tissue. To this end, we immuno‐ stained for Ttr, Toxoplasma, and GFAP in brain sections from *Lcn2CreERT2;Ai9* mice at 42 dpi and 56 dpi following tamoxifen withdrawal (Figure S9e,f). There was no difference in the total count of *Lcn2CreERT2*+ RAs between either time point per ROI, and the proportion of tdTomato+ cells colocalizing with *GFAP* remained at ~50% (Figure S9c,d). Expression of Ttr by *Lcn2CreERT2*+ RAs was judged by colocalization with TdTomato reporter (Figure S9e). At 42 dpi, 50% of *Lcn2CreERT2*+ RAs expressed Ttr which significantly increased to 75% of RAs at the tamoxifen‐rested time point (Figure S9f,g). This finding fits with enrichment of the high Ttr‐expressing *Lcn2CreERT2*+ RA Cluster 2 that expanded substantially between 42 and 56 dpi (Figure 4d). We found little evidence to support localization of *Lcn2CreERT2*+ RAs to parasite cysts in general, with only ~20% of all labeled cells in close proximity (< 10 μm) to cysts. However, unexpectedly, there was a threefold increase in Ttr+ *Lcn2CreERT2*+ RAs adjacent to cysts at late chronic 56 versus 42 dpi time points (Figure S9g). This increased association may indicate that the presence of cysts stimulates or sustains a Ttr+ RA phenotype over time. Overall, these findings suggest a novel population of Ttr‐expressing reactive astrocytes that are enriched for glycolytic genes and can be reliably found at the brain vasculature, suggesting a unique role for these RAs in energy metabolism during chronic infection.

## 4 Discussion

Toxoplasma infection induces acute and chronic inflammatory environments for resident CNS cells. Astrocytes respond to infection and contribute to the immune response by releasing cytokines and chemokines, limiting parasite replication via activation of STAT‐1 signaling, and increasing extracellular glutamate concentrations through a diminished capacity for glutamate uptake (Wilson and Hunter 2004; Sofroniew 2015b; Hidano et al. 2016; David et al. 2016; Still et al. 2020; Orchanian et al. 2024). The diverse roles of astrocytes in response to various CNS insults have recently begun to be established (Zamanian et al. 2012; John Lin et al. 2017; Li et al. 2019; Hasel et al. 2021). Understanding heterogeneous astrocyte responses to disease and injury has the potential to not only decipher this complex biology but also reveal new markers for the development of novel transgenic tools and targets for treatment. Our results suggest that not all astrocytes are pre‐programmed to respond in a similar manner to harmful stimuli, but rather that astrocytic responses are orchestrated by several subtypes with unique genetic profiles that are specialized for particular functions, and that each of these evolves over the course of the inflammatory response.

### 4.1 Astrocyte Functional Diversity During Chronic *T. gondii* Infection

Identification of distinct astrocyte subpopulations allowed us to assign diverse putative roles for astrocytes during chronic *T. gondii* infection. When looking at our first dataset of mixed non‐RAs and RAs sorted based on expression of astrocyte cell‐surface antigen 2 (ACSA‐2), which labels the β2 subunit of the astrocyte sodium‐potassium pump (Batiuk et al. 2020), we found a strong reactive and inflammation‐responding cluster (Cluster 1) defined by high expression of *Cxcl10*, *C3*, and IFN‐γ induced genes *Igtp* and *Iigp1* throughout early and chronic infection (Figure 3a–c). Cluster 1 consistently expanded over the course of infection and consisted of 45% of astrocytes during chronic infection, making it the largest cluster among the eight that were identified based on differentially expressed genes (Figure 2d). We speculate that these astrocytes are some of the earliest responders to infection and are responsible for mediation and maintenance of inflammation as part of the adaptive immune response. A second defining chronic infection cluster (Cluster 3) made up approximately 30% of astrocytes and upregulated *Ttr* and *Enpp2* transcripts, which were tightly correlated with vasculature regulation and tissue homeostasis pathways (Figures 2d and 3e,g). These divergent populations suggest that astrocytes respond and evolve to address the changing CNS environment in a functionally heterogenous manner.

### 4.2 Reactive Astrocyte Markers

One limitation of studying astrogliosis is the lack of tools readily available to identify different functional subsets of RAs, as recent work suggests vast heterogeneity (Sofroniew 2015a; Liddelow and Barres 2017; John Lin et al. 2017; Li et al. 2019; Hasel et al. 2021). Due to the scarcity of tools, tracking specific RA pools has been difficult. Identification of specific markers for astrocyte reactivity is opening avenues to investigate RAs in ways not previously possible. Reactive astrocyte‐specific markers can be used not only to label defined subpopulations of RAs but also to generate genetically encoded tools for RA tracking and manipulation. For example, bulk sequencing data identified *Lcn2* as a gene highly upregulated in RAs in various models of neuroinflammation, but negligibly expressed in the healthy adult brain (Zamanian et al. 2012; Lee et al. 2015; Li, Wang, et al. 2023). Using this information, we recently generated an inducible Cre line driven by the *Lcn2* promoter (Agnew‐Svoboda et al. 2022). In the present study, *Lcn2* was confirmed by single cell sequencing to be strongly upregulated during Toxoplasma‐induced neuroinflammation, defining a subset of RAs. Therefore, we used the *Lcn2CreERT2* mice to follow and characterize this subpopulation of RAs during disease progression, highlighting the utility of such tools. Thus, in addition to allowing for direct comparisons between cell populations across a wide array of inflammatory disease models, single cell RNA seq data can be used to generate RA subtype‐specific genetic tools. Future experiments utilizing fluorescent labeling and live imaging can generate new insights into possible functional roles of RAs, such as migration and proliferation, across multiple diseases and CNS injuries (Escartin et al. 2021). One caveat to this tool is that we are only able to investigate a subset of RAs defined by *Lcn2* expression. Although it allows a more in‐depth look at some RAs, it does not permit analysis of the overall range of RA heterogeneity (Agnew‐Svoboda et al. 2022). Therefore, *Lcn2CreERT2* mice are a great complementary tool to sequencing and sorting approaches based on other astrocyte marker expression such as ACSA‐2. Thus, although Lcn2 RAs are diverse following Toxoplasma infection, it is likely that astrocyte heterogeneity remains underestimated.

### 4.3 Lcn2CreERT2+ Reactive Astrocyte Loss and Gain of Function During Chronic Toxoplasma Infection

The *Lcn2CreERT2+* RA clusters were similar to populations observed in our initial experiments based on sorting for ASCA‐2, revealing an immune‐responsive pool in Cluster 1 defined by the upregulation of *Ccl5, Cxcl9*, and *Cxcl10* during acute and chronic infection, and a subset in Cluster 2 characterized by upregulation of *Ttr* and *Ptgds* (Figure 4a). Our findings suggest the presence of RAs with sustained protective functions, supported by data showing enrichment of genes associated with anti‐inflammatory mediators, glycolytic activity, and vasculature regulation. This is complemented by other RAs with enrichment of proinflammatory mediators that may contribute to a sustained immune response to contain infection. Key neurotransmitter‐regulating genes, such as GLT‐1, were found to be significantly reduced in *Lcn2CreERT2+* astrocyte Clusters 1–4 (Figure 5e). The two largest populations, Clusters 1 and 2, initially identified as immune regulatory and infection responsive, respectively, were surprisingly low in glutamate transporter transcript expression. Cluster 5 was enriched for glutamate transporter transcripts in both datasets but dwindled to only 3% of the astrocyte population over the course of infection. It is known that not all astrocytes express GLT‐1 (*Slc1a2*) and even fewer express GLAST (*Slc1a3*) (Rimmele and Rosenberg 2016), which may account for the heterogeneity observed at baseline in uninfected naïve mice. GLT‐1 expression also differs between astrocytic subcompartments such as the peri‐synaptic astrocytic process (PAP), near synapses, or near the vasculature (de Vivo et al. 2010). Additionally, scRNA sequencing is tuned to detect highly expressed genes, but the depth of sequencing is not as great as for bulk RNA sequencing. Therefore, the lack of GLT‐1 transcripts in this data set might be indicative of lower expression relative to other clusters rather than an absence of expression. Overall, these findings are in line with our previous work identifying localized GLT‐1 protein loss (David et al. 2016) but support the concept that not all astrocytes are affected and may be transient within a population (Figure 5). Indeed, recovery of GLT‐1 expression may explain the relative subclinical nature of Toxoplasma chronic infection.

We speculate that the changes occurring in RAs traditionally considered detrimental, that is, loss of glutamate uptake and increased inflammation, are a necessary part of the immune response to contain the predominant insult, which in this case is infection. During *T. gondii* infection there is a notable increase in genes associated with the immune response, metabolism, and vasculature control, while expression of genes typically involved in homeostatic functions such as regulation of neurotransmitters are significantly reduced. However, increasing extracellular glutamate concentrations may help to facilitate and coordinate the acute immune response within brain tissue by providing metabolic support to immune responsive cells. Astrocytes are known for their metabolic supportive functions (Zhang et al. 2023). Immune cells additionally increase expression of metabotropic glutamate receptors (Vizcarra et al. 2023). Diminished glutamate uptake in discrete brain regions may attract lymphocytes to areas of tissue pathology. Indeed, mGluR signaling on T cells optimizes their production of the protective cytokine IFN‐γ (Vizcarra et al. 2023). Therefore, its availability may be critical in the local CNS to fight infection. This ultimately could be revealing limitations in the number of functions astrocytes can perform simultaneously: as they physically and functionally respond to the disrupted environment by becoming immune‐responsive cells, they transiently or chronically lose their ability to maintain regulatory roles. Whether this is done at the expense of metabolic factors, or a predetermined pathway astrocytes obtain during maturation, is unknown.

### 4.4 RA Heterogeneity Is a Dynamic Process During Infection

Gaining insight into the dynamics of astrogliosis over time and among subpopulations adds to the growing body of literature on astrocyte diversity and heterogeneity (Escartin et al. 2021). Some populations, for example, might largely resolve over time, while others may evolve into a chronic immunoreactive subtype, taking on new characteristics while losing others. Within *Lcn2CreERT2*+ RAs, we observed a sustained neuroimmune population (Cluster 1), which persisted 2 weeks after cessation of Cre induction, an example of a subset of astrocytes that does not return to their transient state. On the other hand, *Lcn2CreERT2*+ Cluster 5 appeared to more closely resemble naïve and acutely responding astrocytes (Figures 4c and 5) when tracked longitudinally between 42 and 56 dpi. Interestingly, *Lcn2CreERT2*+ Cluster 5 (enriched for *Slc1a2*; Figure 5e,f) resembled transcriptional patterns similar to Cluster 5 in the naïve brain (Figures 3a and S3g), suggesting some diminishment of chronic inflammation in favor of recovery of homeostatic astrocyte functions.

Our data suggest minimal resolution of reactive gliosis following long‐term *T. gondii* infection based on the re‐emergence of the small *Lcn2CreERT2*+ Cluster 5 late in infection. It is important to note that the experiments in the current study were performed in *C57BL/6* mice which mimic the pathology and progression of Toxoplasma infection in susceptible individuals, eventually ending in severe symptoms and Toxoplasma reactivation (Tanaka et al. 2013; David et al. 2016; Bergersen et al. 2021). This immune environment resembles several aspects of acute innate infection and can be fatal in late chronically infected mice. Performing these experiments in a more resilient mouse strain such as *BALB/c* might reveal more sustained resolution of inflammation based on the relative proportions of astrocyte subsets. *BALB/c* mice establish high resistance to the parasite and could be a useful genetic background to determine which particular RA populations diminish or increase over time when the brain environment stabilizes into maintenance mode for parasite containment (Bergersen et al. 2021). Unlike Toxoplasma infection, which remains in the brain throughout life, we would expect either most RAs to revert back into non‐reactive states once inflammation resolves, as we recently observed in the case of systemic LPS (Agnew‐Svoboda et al. 2022), or prevalence of reactive astrocyte subsets that may be more neuroprotective or supportive in nature. Future studies investigating how *Lcn2CreERT2*+ RA populations change over time could be useful in identifying permanently vigilant astrocytes versus those that acutely respond but then largely recover to a non‐reactive state with classical supportive functions.

### 4.5 Transthyretin RAs

*Transthyretin's* role in astrocytes has been largely overlooked, as many studies have focused on neuron‐derived *Ttr* modulating glial energy metabolism (Liz et al. 2020; Zawiślak et al. 2017). Accumulating evidence highlights the diverse roles *Ttr* plays in the CNS (Santos et al. 2010; Nunes et al. 2013; Rawat et al. 2022; Li, Xu, et al. 2023). One specific protective role of *Ttr* during chronic neuroinflammation is its binding to Aβ to facilitate clearance (Alemi et al. 2016; Gião et al. 2020). *Lcn2Cre* RA Cluster 2 enriched for *Ttr* also expressed glycolytic genes such as *Gpi1*, *Hk1*, and *Gskb1* but displayed low expression of glucose and monocarboxylate transporters, suggesting *Ttr*‐enriched astrocytes have a different role than storing glucose as compared to other astrocyte clusters (Figure 6d,e). Elevated levels of *Ldhb* in *Ttr*‐enriched RAs (Figure 6d,e) could be a response to Toxoplasma infection to meet increased metabolic demand through conversion of lactate into pyruvate (Bittar et al. 1996; Ross et al. 2010). Typically, astrocytes are glycolytic cells, but this metabolic shift from Ldha to Ldhb is consistent with energy generation through the TCA cycle rather than glycolysis, as pyruvate can enter the TCA cycle. Astrocytes typically release lactate to meet the metabolic requirements of highly active neurons through the astrocyte‐neuron lactate shuttle (ANLS) (Pellerin and Magistretti 2012). The capability of the metabolic shift Ttr‐rich astrocytes undergo is unusual but, at the same time, an effective alternative way to generate energy, especially in close proximity to cysts where *Ttr*‐expressing astrocytes are prevalent (Figure S9e–f).

Progression of neuroinflammation during Toxoplasma infection is worsened by a dysfunction in vasculature and reduction of angiogenesis in infected mice (Estato et al. 2018). The roles of astrocytes in regulating the BBB and vasculature function are well‐documented and involve bidirectional communication with endothelial cells and pericytes (Alvarez et al. 2011; Puebla et al. 2022). Many studies have assessed BBB permeability and the potential responses of astrocytes in brain injury and disease, including epilepsy, brain injury, and infection (Mathiisen et al. 2010; David et al. 2016; Nippert et al. 2018). An additional important aspect of regulating vasculature function is the formation of new vessels (Williamson et al. 2021). Angiogenesis appears to be essential to combat neurodegeneration during chronic neuroinflammatory disease (Puebla et al. 2022; La Mendola et al. 2022). It would be worthwhile to probe deeper into how this process can be initiated and maintained in different disease states and if delivery of oxygen and glucose is locally compromised. Transthyretin's association with angiogenesis is an under‐researched area. Future studies can help identify mechanisms by which astrocytes regulate angiogenesis and the BBB during neuroinflammation and determine if widespread CNS insult recruits astrocytes to the vasculature either to help regulate homeostasis or facilitate the immune response.

Numerous subtypes of astrocytes have been defined at the anatomical and transcriptional levels in the healthy brain (Tabata 2015; Li et al. 2019; Hasel et al. 2021). Following brain injury or infection, astrocyte reactivity has been traditionally defined based on increased intensity of GFAP staining by immunohistochemistry. Despite widespread upregulation of GFAP during *Toxoplasma gondii* infection, astrocytes do not respond homogeneously, similar to the heterogeneity that exists prior to infection. Whole brain samples were used due to the difficulty in retrieving sufficient numbers of RAs from sorted dissected anatomical regions. Therefore, some of the observed heterogeneity between reactive astrocytes may reflect the natural diversity of astrocytes under resting conditions between different brain regions. In addition, as with all ex vivo sampling, our analysis may be missing subpopulations of cells that are particularly difficult to extract due to tissue structure or susceptibility to cell death, thereby biasing the data toward the hardiest of astrocytes. These data are now available for further validation through spatial and in situ techniques to determine their phenotype and location within the brain.

In summary, we have combined sequencing of astrocytes with selective scRNAseq analysis of Lcn2Cre‐expressing RAs to identify unique RA subpopulations during infection by the protozoan parasite, *T. gondii*. Our findings indicate a dynamic, non‐homogeneous astrocyte population comprised of multiple subtypes that are enriched with specific functions and change in prevalence over the course of infection. In particular, we highlight a prominent subset that strongly upregulates expression of transthyretin, suggesting an important role for RAs in regulation of CNS metabolism and the vasculature as a component of the immune response in the brain. Understanding mechanisms that underlie RA function and heterogeneity will be crucial in delineating therapeutic approaches for the treatment of chronic inflammation‐related disease.

## Author Contributions

Z.A.F. and E.H.W. designed the experiments. Z.A.F. and J.L.M. performed the experiments. Z.A.F. analyzed the data. T.A.F., M.M.R., and E.H.W. conceived the project; Z.A.F. and E.H.W. interpreted the data; and Z.A.F., T.A.F., and E.H.W. wrote the manuscript. Z.A.F., J.L.M., A.U., T.U., T.A.F., M.M.R., and E.H.W. edited the manuscript.

## Conflicts of Interest

The authors declare no conflicts of interest.

## Supporting information


**Data S1** Supporting Information.

**Data S2** Supporting Information 1.

**Data S3** Supporting Information 2.

**Data S4** Supporting Information 3.

**Data S5** Supporting Information 4.

**Data S6** Supporting Information 5.

**Data S7** Supporting Information 6.

**Data S8** Supporting Information 7.

**Data S9** Supporting Information 8.

**Data S10** Supporting Information 9.

**Data S11** Supporting Information 10.

**Data S12** Supporting Information 11.

**Data S13** Supporting Information 12.

**Data S14** Supporting Information 13.

## Data Availability

The raw BMSC, ACSA‐2 sorted, and *Lcn2CreERT2*+ sorted scRNAseq files for the characterization and analysis of samples are deposited for public use in the Gene Expression Omnibus (GEO) and GenBank. These sequence data have been submitted to the databases under accession number GSE274474.
