# Supplementary material for: Diverse Subpopulations of Reactive Astrocytes Following Chronic Toxoplasma Infection
Source: Glia. 2025 Jul 9;73(10):2003–24. doi: 10.1002/glia.70053 (PMC12244103; doi:10.1002/glia.70053)
Supplement: Supplementary file 1 — Data S1 Supporting Information. [file GLIA-73-2003-s012.pptx]

## Slide 1
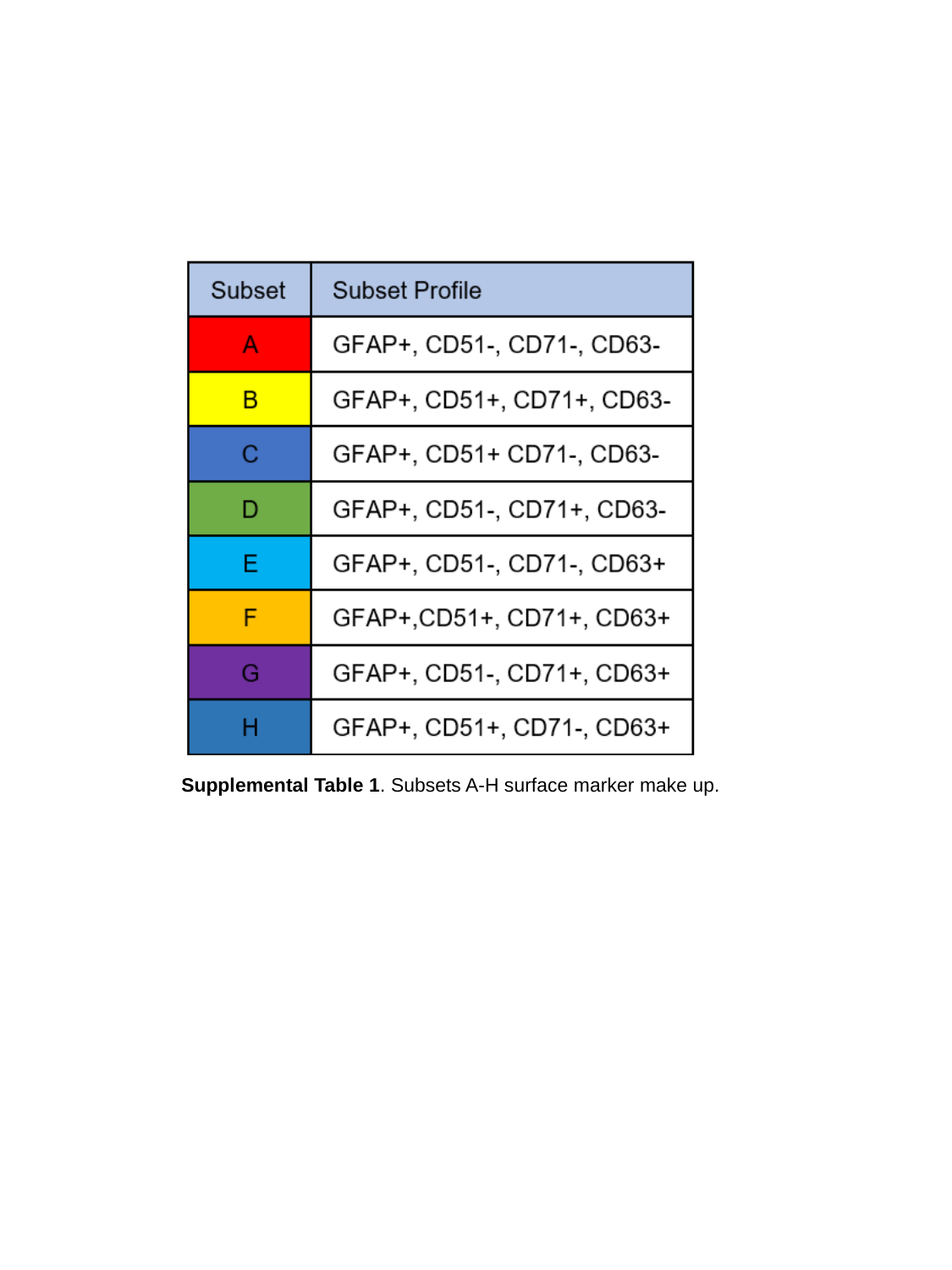

Supplemental Table 1. Subsets A-H surface marker make up.

## Slide 2
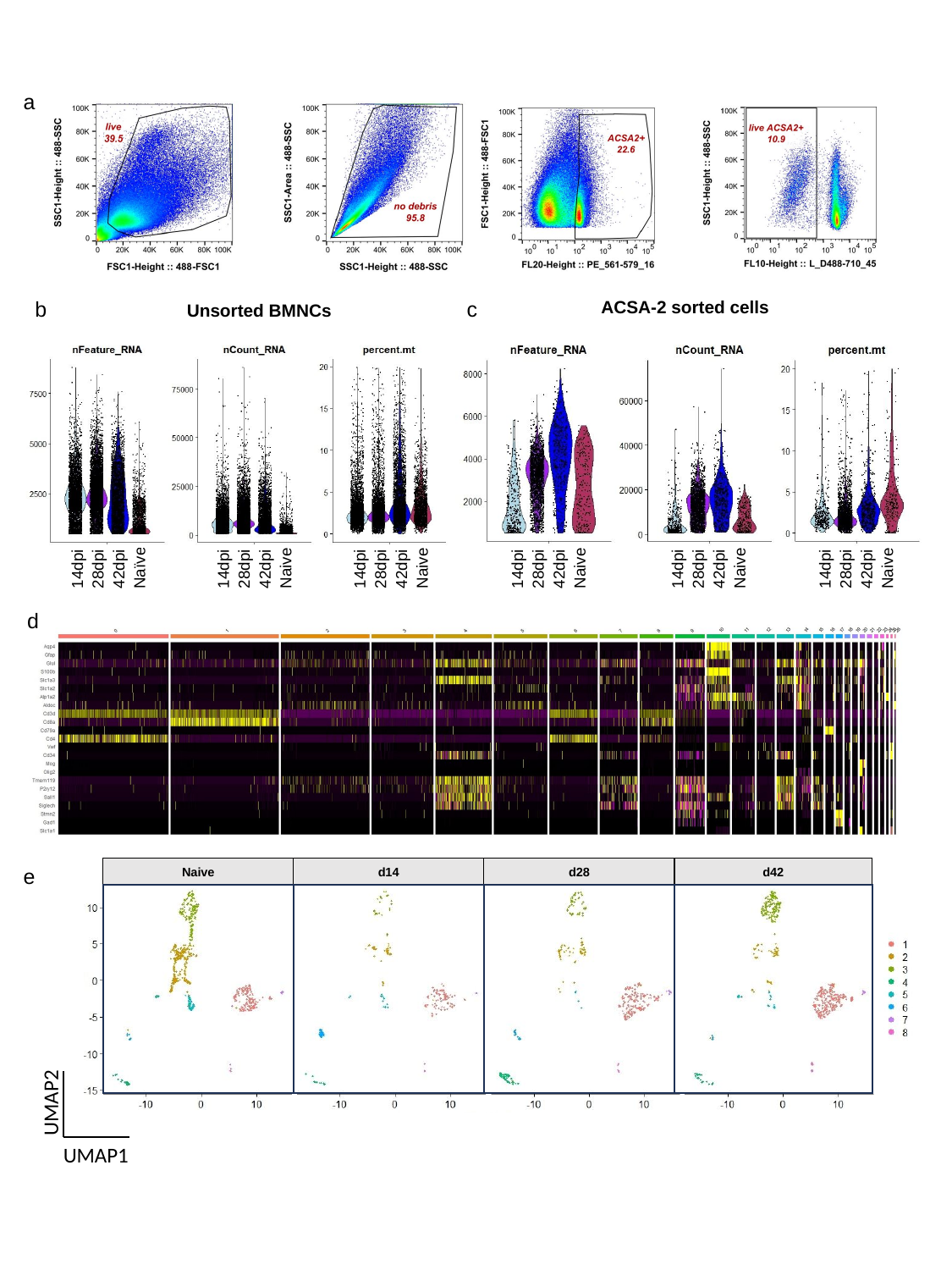

a
b
c
ACSA-2 sorted cells
Unsorted BMNCs
14dpi
28dpi
42dpi
Naive
14dpi
28dpi
42dpi
Naive
14dpi
28dpi
42dpi
Naive
14dpi
28dpi
42dpi
Naive
14dpi
28dpi
42dpi
Naïve
14dpi
28dpi
42dpi
Naive
d
e
d28
d42
Naive
d14
UMAP2
UMAP1

## Slide 3
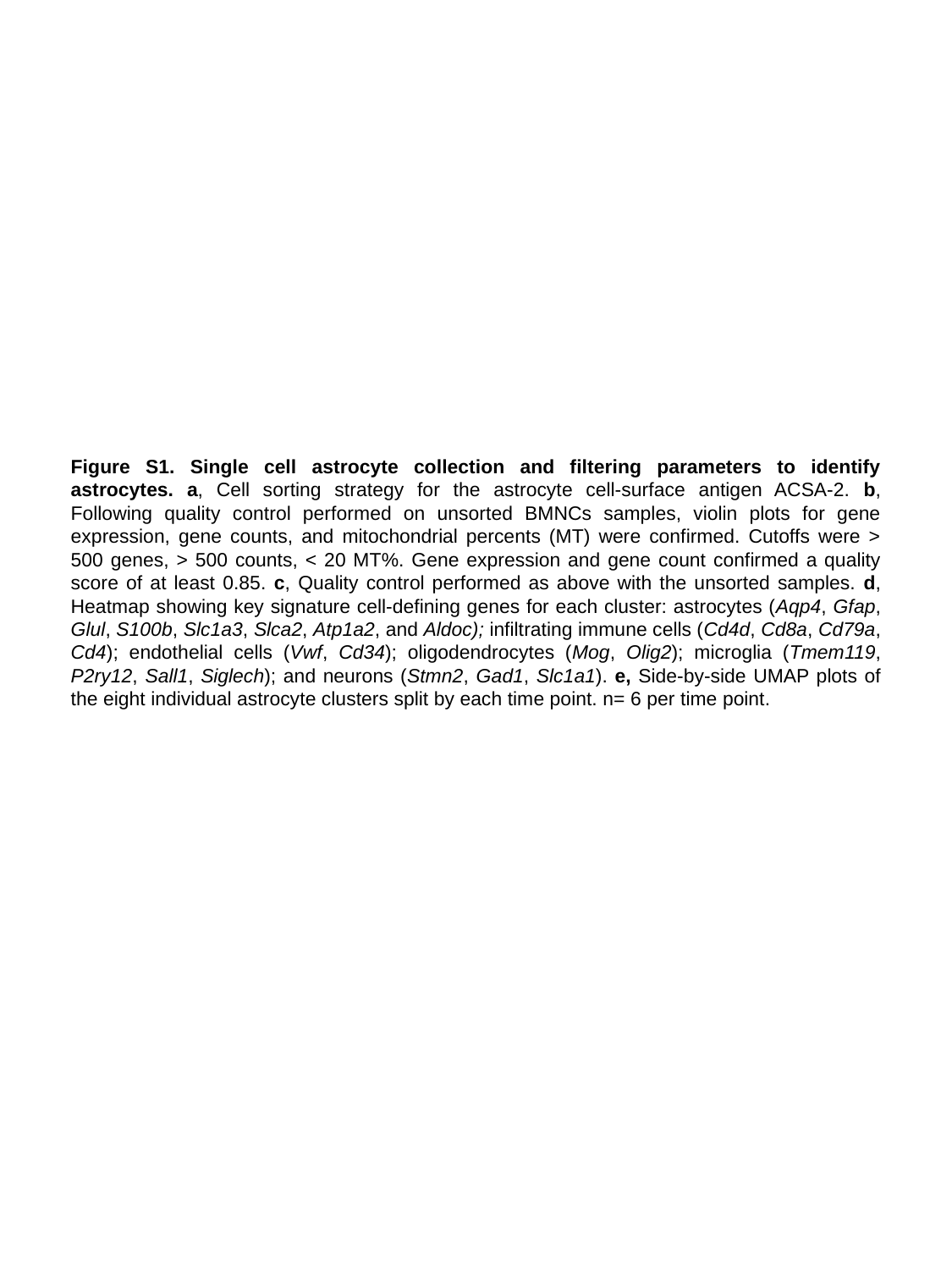

Figure S1. Single cell astrocyte collection and filtering parameters to identify astrocytes. a, Cell sorting strategy for the astrocyte cell-surface antigen ACSA-2. b, Following quality control performed on unsorted BMNCs samples, violin plots for gene expression, gene counts, and mitochondrial percents (MT) were confirmed. Cutoffs were > 500 genes, > 500 counts, < 20 MT%. Gene expression and gene count confirmed a quality score of at least 0.85. c, Quality control performed as above with the unsorted samples. d, Heatmap showing key signature cell-defining genes for each cluster: astrocytes (Aqp4, Gfap, Glul, S100b, Slc1a3, Slca2, Atp1a2, and Aldoc); infiltrating immune cells (Cd4d, Cd8a, Cd79a, Cd4); endothelial cells (Vwf, Cd34); oligodendrocytes (Mog, Olig2); microglia (Tmem119, P2ry12, Sall1, Siglech); and neurons (Stmn2, Gad1, Slc1a1). e, Side-by-side UMAP plots of the eight individual astrocyte clusters split by each time point. n= 6 per time point.

## Slide 4
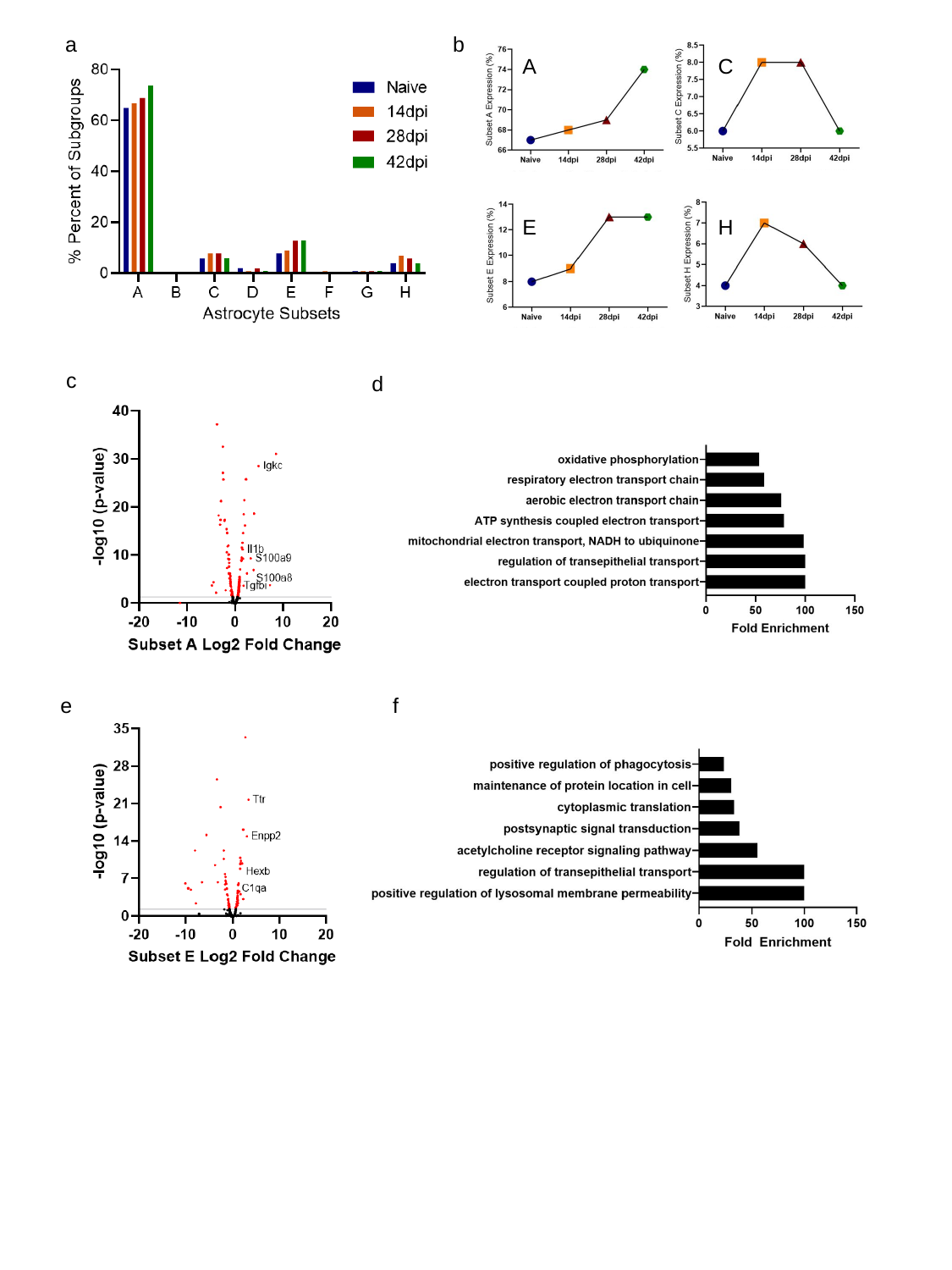

a
b
A
C
H
E
c
d
e
f

## Slide 5
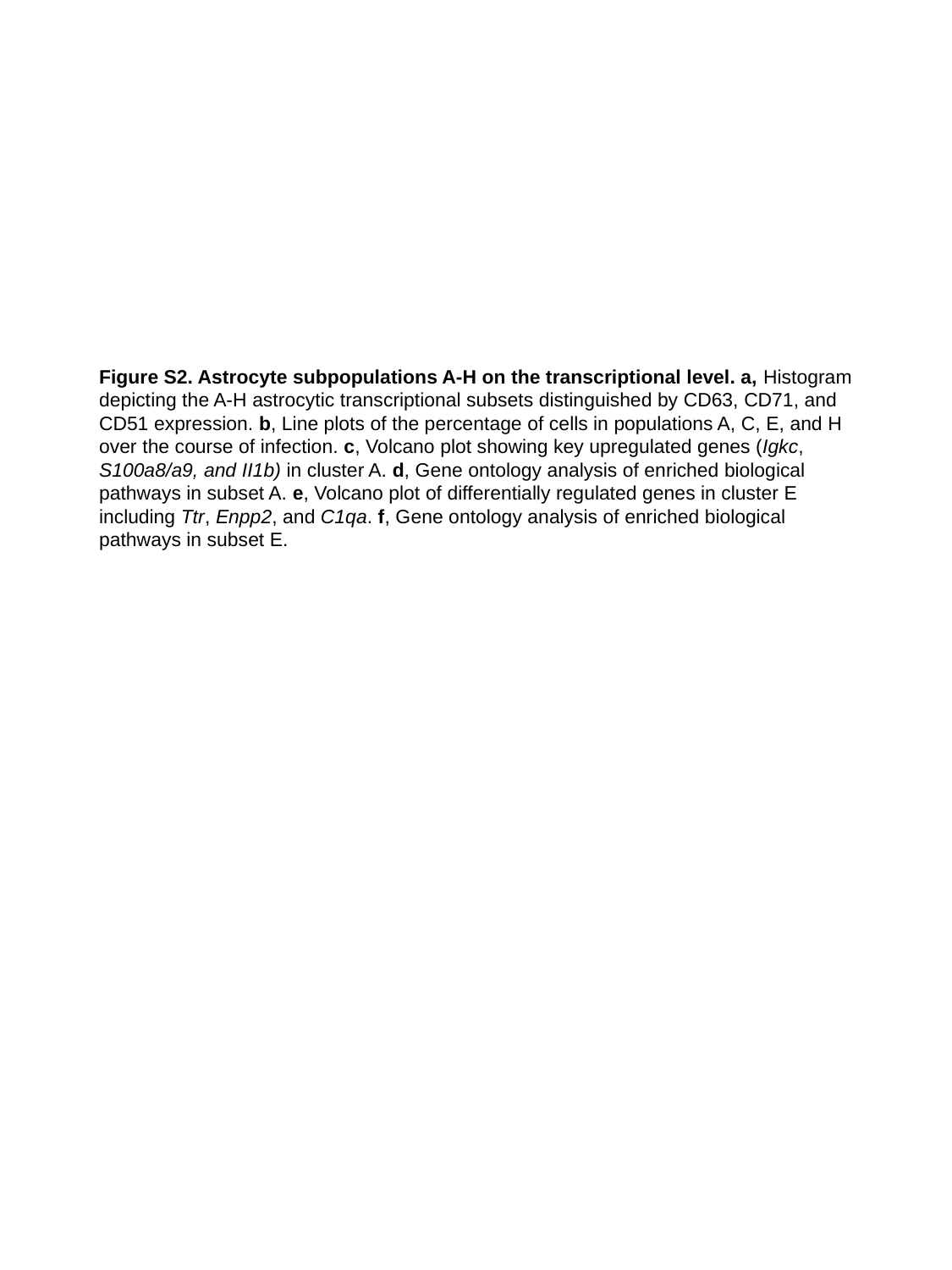

Figure S2. Astrocyte subpopulations A-H on the transcriptional level. a, Histogram depicting the A-H astrocytic transcriptional subsets distinguished by CD63, CD71, and CD51 expression. b, Line plots of the percentage of cells in populations A, C, E, and H over the course of infection. c, Volcano plot showing key upregulated genes (Igkc, S100a8/a9, and II1b) in cluster A. d, Gene ontology analysis of enriched biological pathways in subset A. e, Volcano plot of differentially regulated genes in cluster E including Ttr, Enpp2, and C1qa. f, Gene ontology analysis of enriched biological pathways in subset E.

## Slide 6
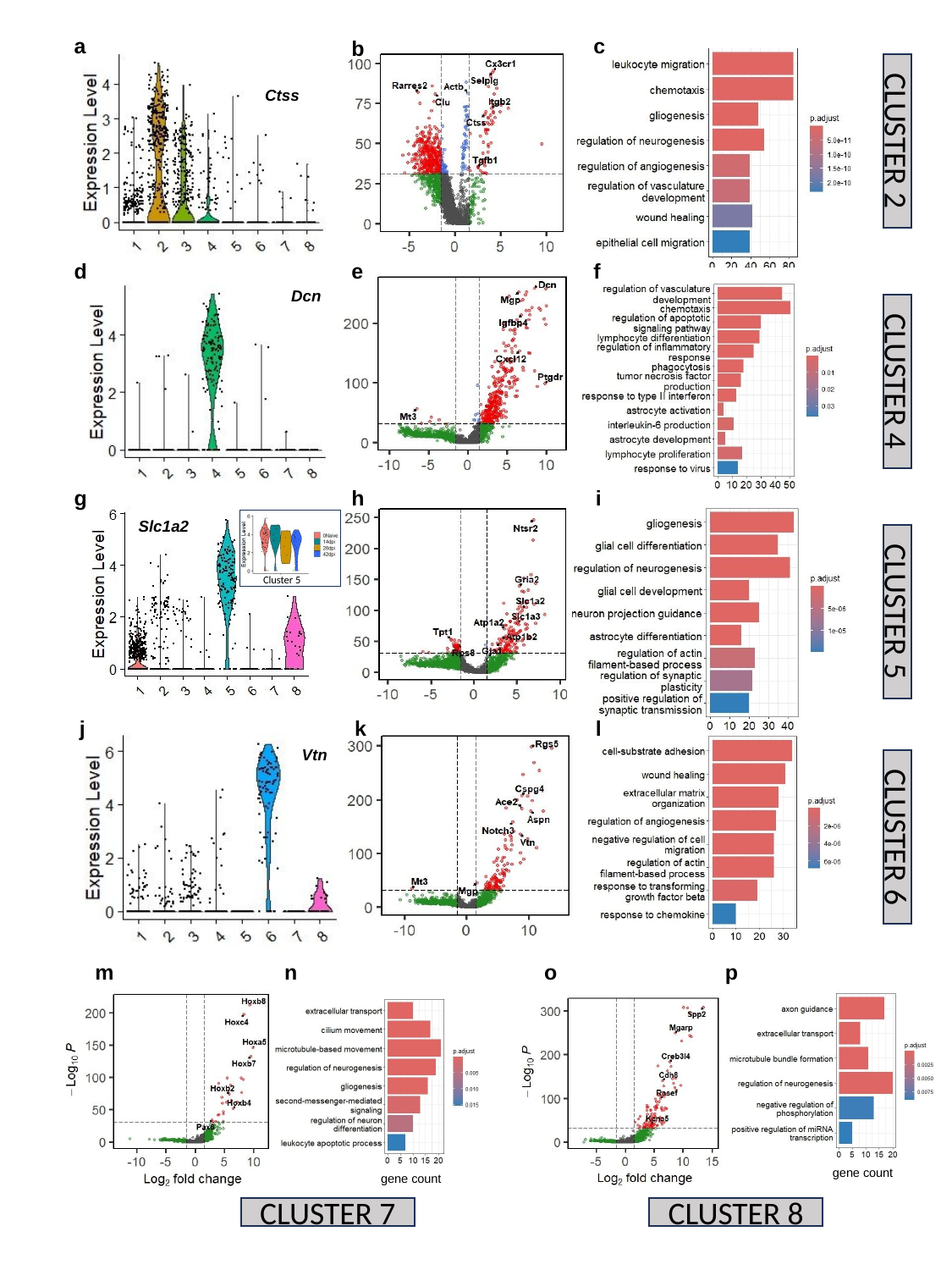

a
c
b
Ctss
CLUSTER 2
d
e
f
Dcn
CLUSTER 4
g
h
i
gene count
Slc1a2
Cluster 5
CLUSTER 5
j
k
l
Vtn
CLUSTER 6
m
n
o
p
gene count
gene count
CLUSTER 7
CLUSTER 8

## Slide 7
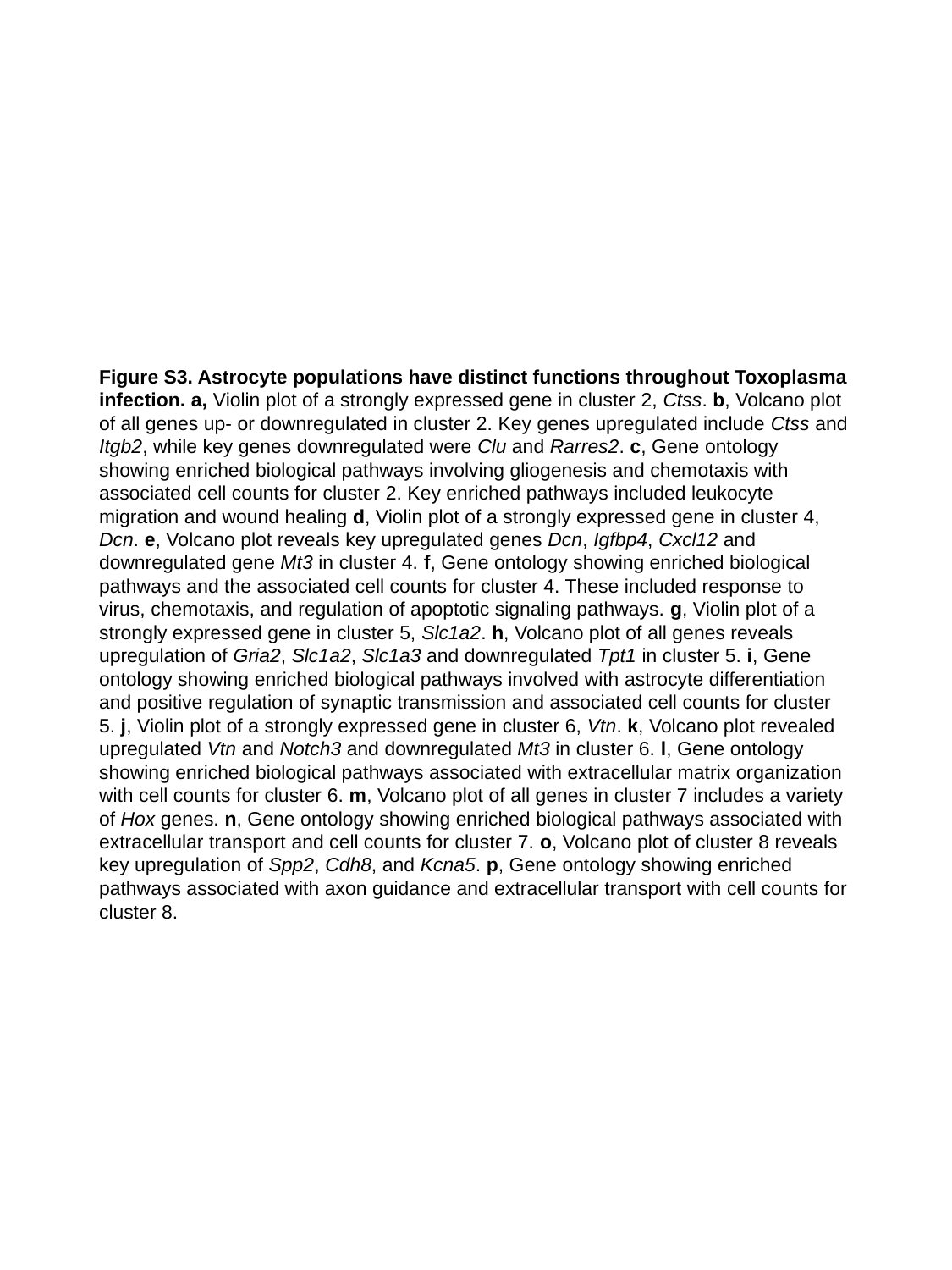

Figure S3. Astrocyte populations have distinct functions throughout Toxoplasma infection. a, Violin plot of a strongly expressed gene in cluster 2, Ctss. b, Volcano plot of all genes up- or downregulated in cluster 2. Key genes upregulated include Ctss and Itgb2, while key genes downregulated were Clu and Rarres2. c, Gene ontology showing enriched biological pathways involving gliogenesis and chemotaxis with associated cell counts for cluster 2. Key enriched pathways included leukocyte migration and wound healing d, Violin plot of a strongly expressed gene in cluster 4, Dcn. e, Volcano plot reveals key upregulated genes Dcn, Igfbp4, Cxcl12 and downregulated gene Mt3 in cluster 4. f, Gene ontology showing enriched biological pathways and the associated cell counts for cluster 4. These included response to virus, chemotaxis, and regulation of apoptotic signaling pathways. g, Violin plot of a strongly expressed gene in cluster 5, Slc1a2. h, Volcano plot of all genes reveals upregulation of Gria2, Slc1a2, Slc1a3 and downregulated Tpt1 in cluster 5. i, Gene ontology showing enriched biological pathways involved with astrocyte differentiation and positive regulation of synaptic transmission and associated cell counts for cluster 5. j, Violin plot of a strongly expressed gene in cluster 6, Vtn. k, Volcano plot revealed upregulated Vtn and Notch3 and downregulated Mt3 in cluster 6. l, Gene ontology showing enriched biological pathways associated with extracellular matrix organization with cell counts for cluster 6. m, Volcano plot of all genes in cluster 7 includes a variety of Hox genes. n, Gene ontology showing enriched biological pathways associated with extracellular transport and cell counts for cluster 7. o, Volcano plot of cluster 8 reveals key upregulation of Spp2, Cdh8, and Kcna5. p, Gene ontology showing enriched pathways associated with axon guidance and extracellular transport with cell counts for cluster 8.

## Slide 8
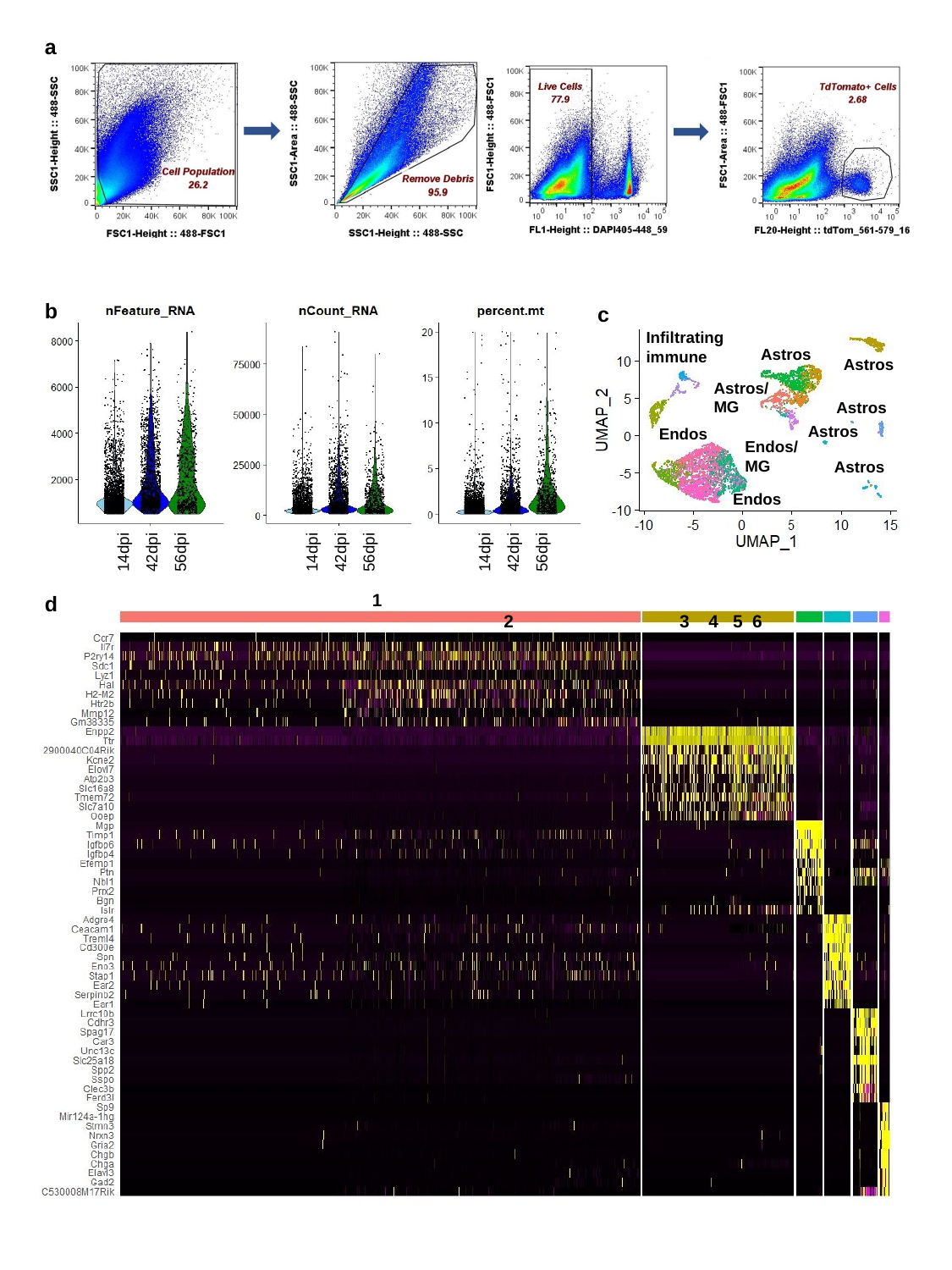

a
b
c
Infiltrating immune
Astros
Astros
Astros/MG
Astros
Astros
Endos
Endos/MG
Astros
Endos
14dpi
42dpi
56dpi
14dpi
42dpi
56dpi
14dpi
42dpi
56dpi
1					 2	 3 4 5 6
d

## Slide 9
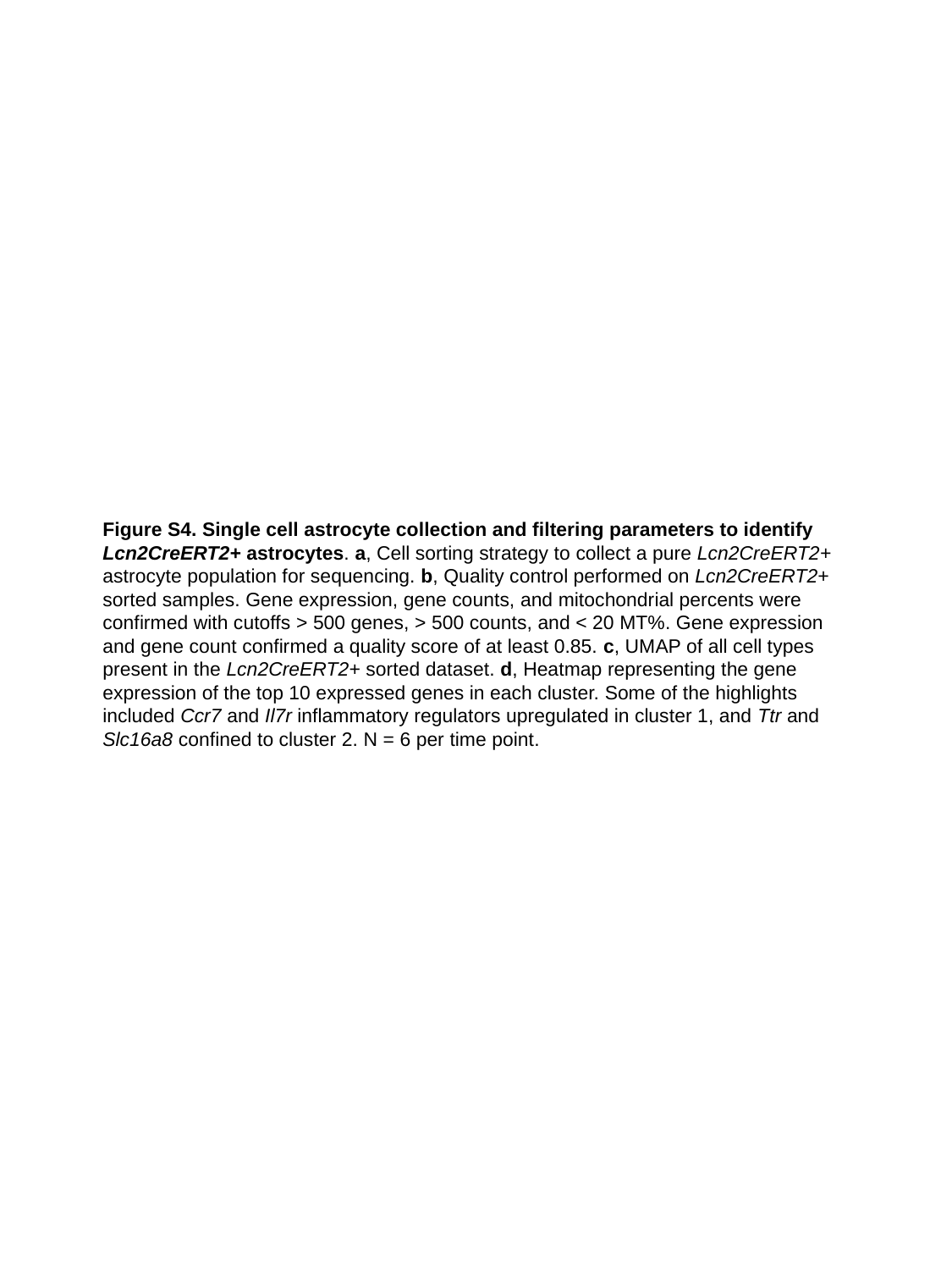

Figure S4. Single cell astrocyte collection and filtering parameters to identify Lcn2CreERT2+ astrocytes. a, Cell sorting strategy to collect a pure Lcn2CreERT2+ astrocyte population for sequencing. b, Quality control performed on Lcn2CreERT2+ sorted samples. Gene expression, gene counts, and mitochondrial percents were confirmed with cutoffs > 500 genes, > 500 counts, and < 20 MT%. Gene expression and gene count confirmed a quality score of at least 0.85. c, UMAP of all cell types present in the Lcn2CreERT2+ sorted dataset. d, Heatmap representing the gene expression of the top 10 expressed genes in each cluster. Some of the highlights included Ccr7 and Il7r inflammatory regulators upregulated in cluster 1, and Ttr and Slc16a8 confined to cluster 2. N = 6 per time point.

## Slide 10
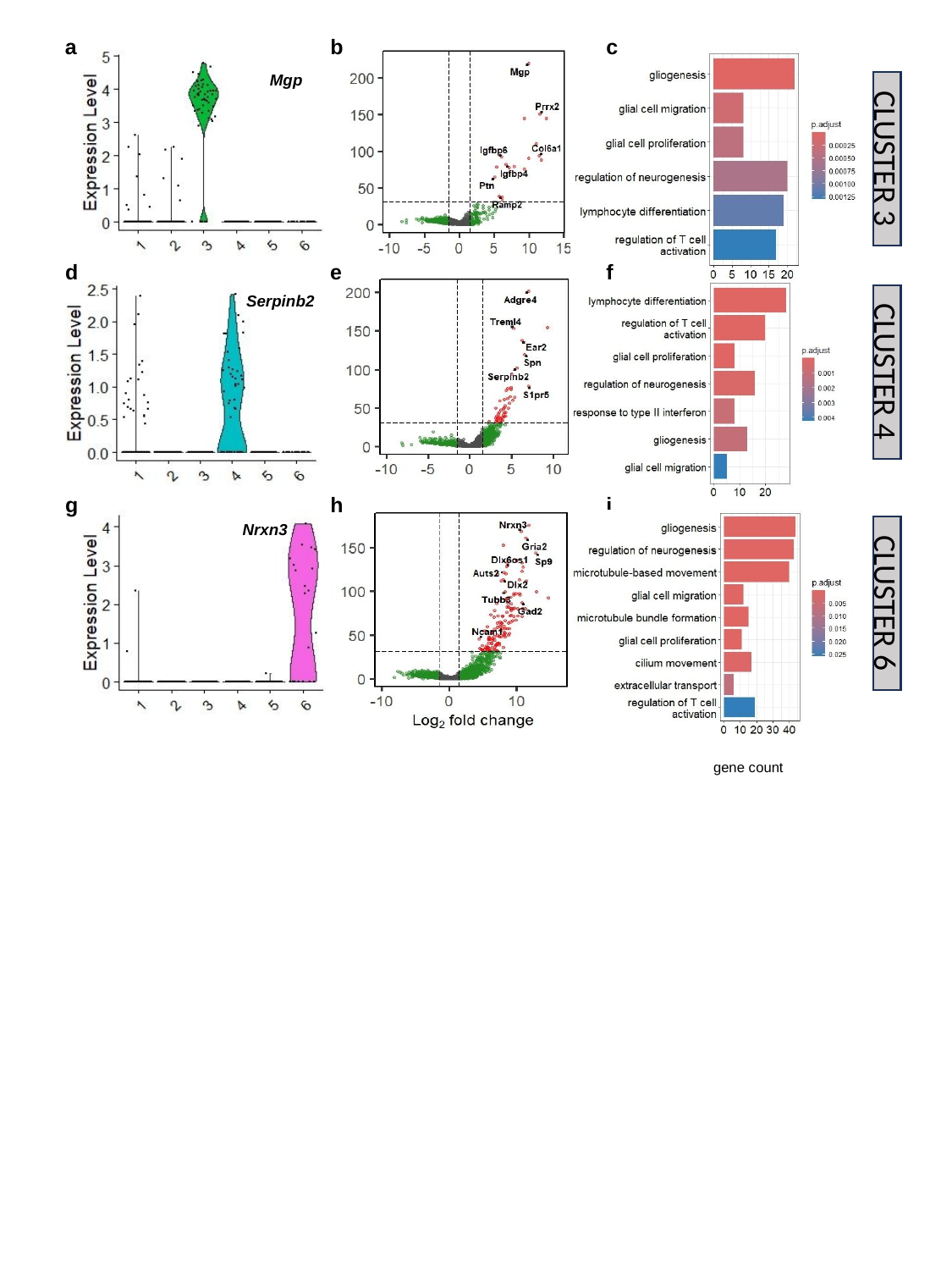

a
b
c
Mgp
CLUSTER 3
d
e
f
Serpinb2
CLUSTER 4
g
h
i
Nrxn3
CLUSTER 6
gene count

## Slide 11
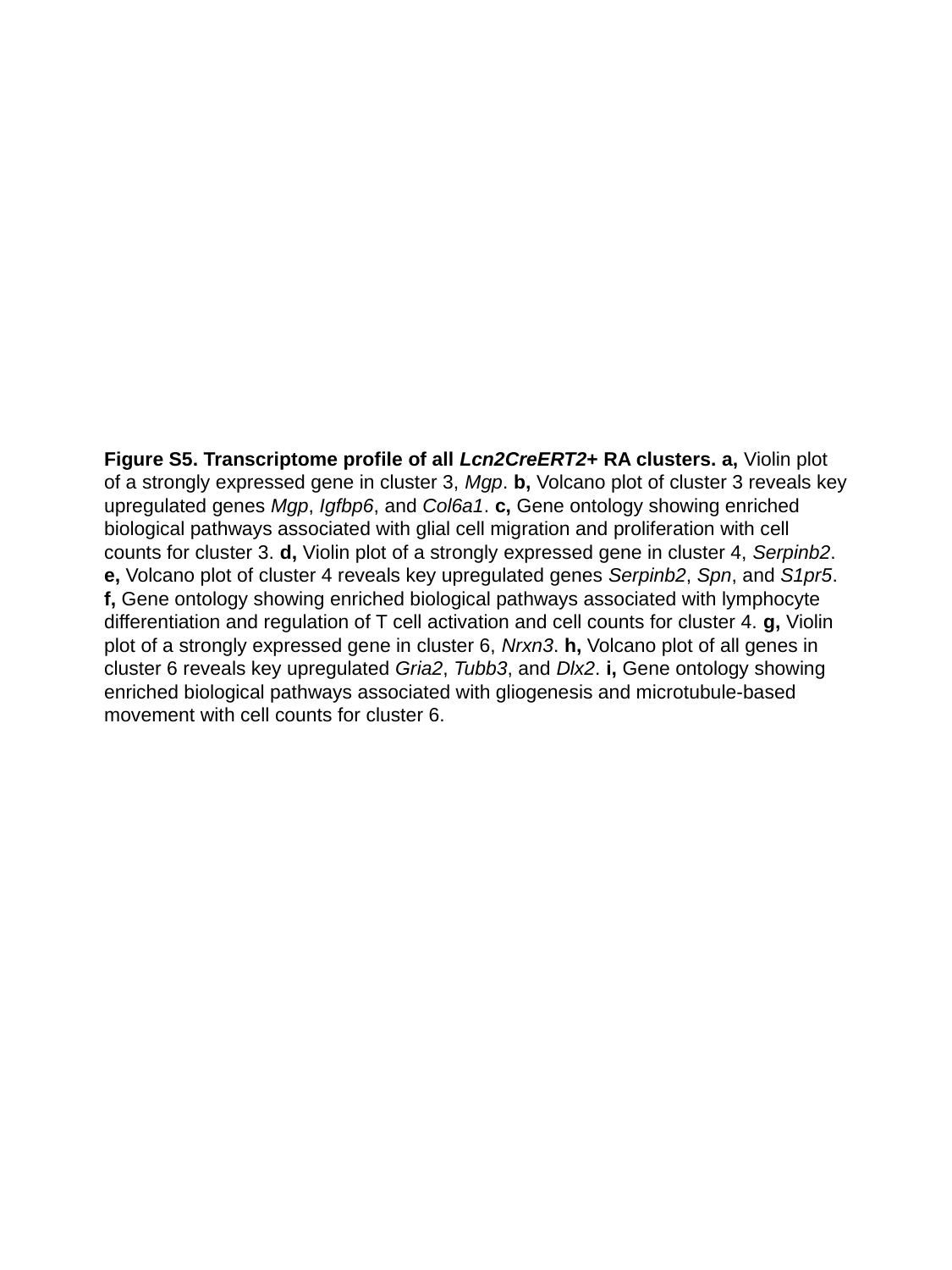

Figure S5. Transcriptome profile of all Lcn2CreERT2+ RA clusters. a, Violin plot of a strongly expressed gene in cluster 3, Mgp. b, Volcano plot of cluster 3 reveals key upregulated genes Mgp, Igfbp6, and Col6a1. c, Gene ontology showing enriched biological pathways associated with glial cell migration and proliferation with cell counts for cluster 3. d, Violin plot of a strongly expressed gene in cluster 4, Serpinb2. e, Volcano plot of cluster 4 reveals key upregulated genes Serpinb2, Spn, and S1pr5. f, Gene ontology showing enriched biological pathways associated with lymphocyte differentiation and regulation of T cell activation and cell counts for cluster 4. g, Violin plot of a strongly expressed gene in cluster 6, Nrxn3. h, Volcano plot of all genes in cluster 6 reveals key upregulated Gria2, Tubb3, and Dlx2. i, Gene ontology showing enriched biological pathways associated with gliogenesis and microtubule-based movement with cell counts for cluster 6.

## Slide 12
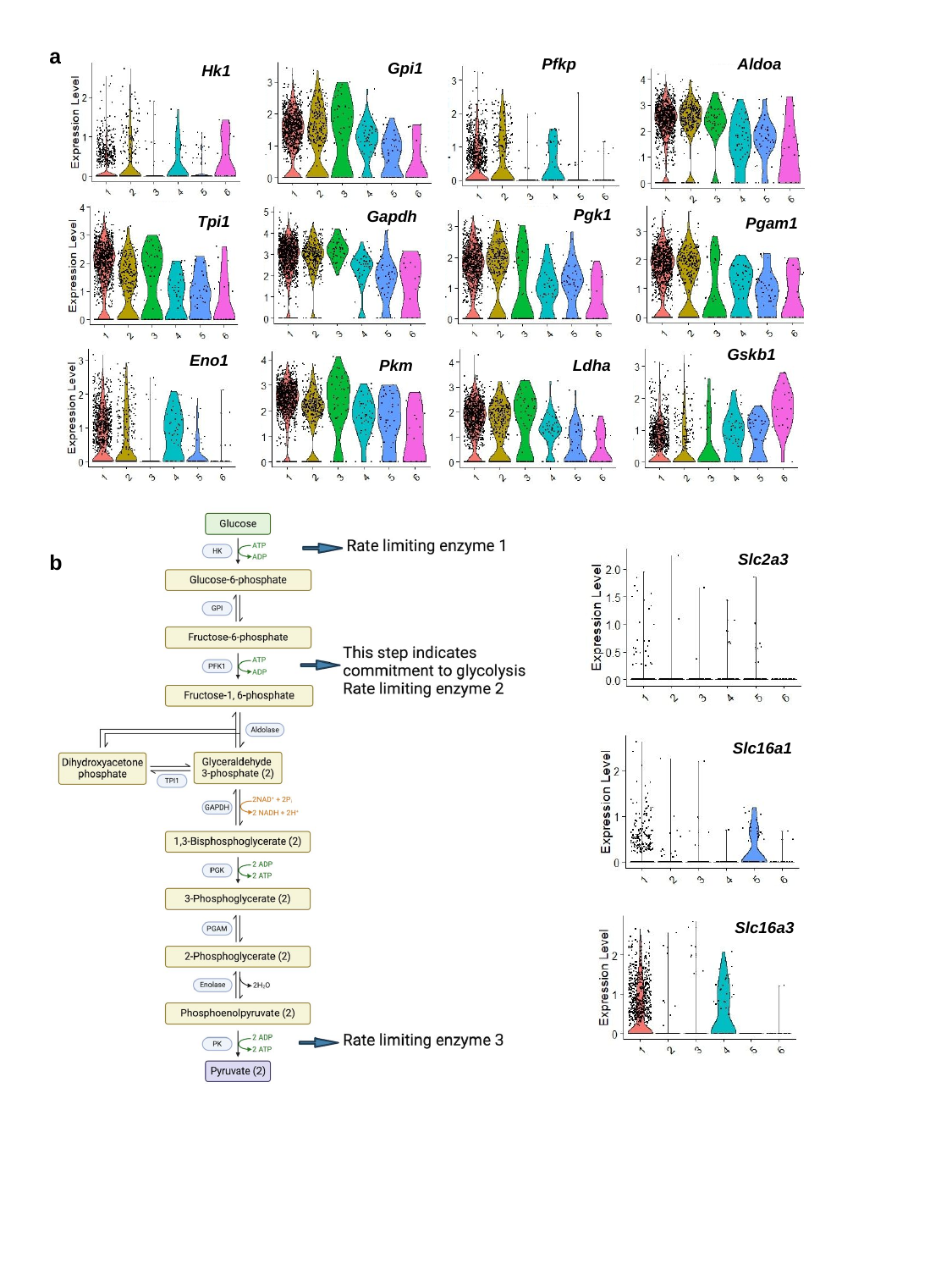

a
Pfkp
Aldoa
Gpi1
Hk1
Pgk1
Gapdh
Tpi1
Pgam1
Gskb1
Eno1
Pkm
Ldha
b
Slc2a3
Slc16a1
Slc16a3

## Slide 13
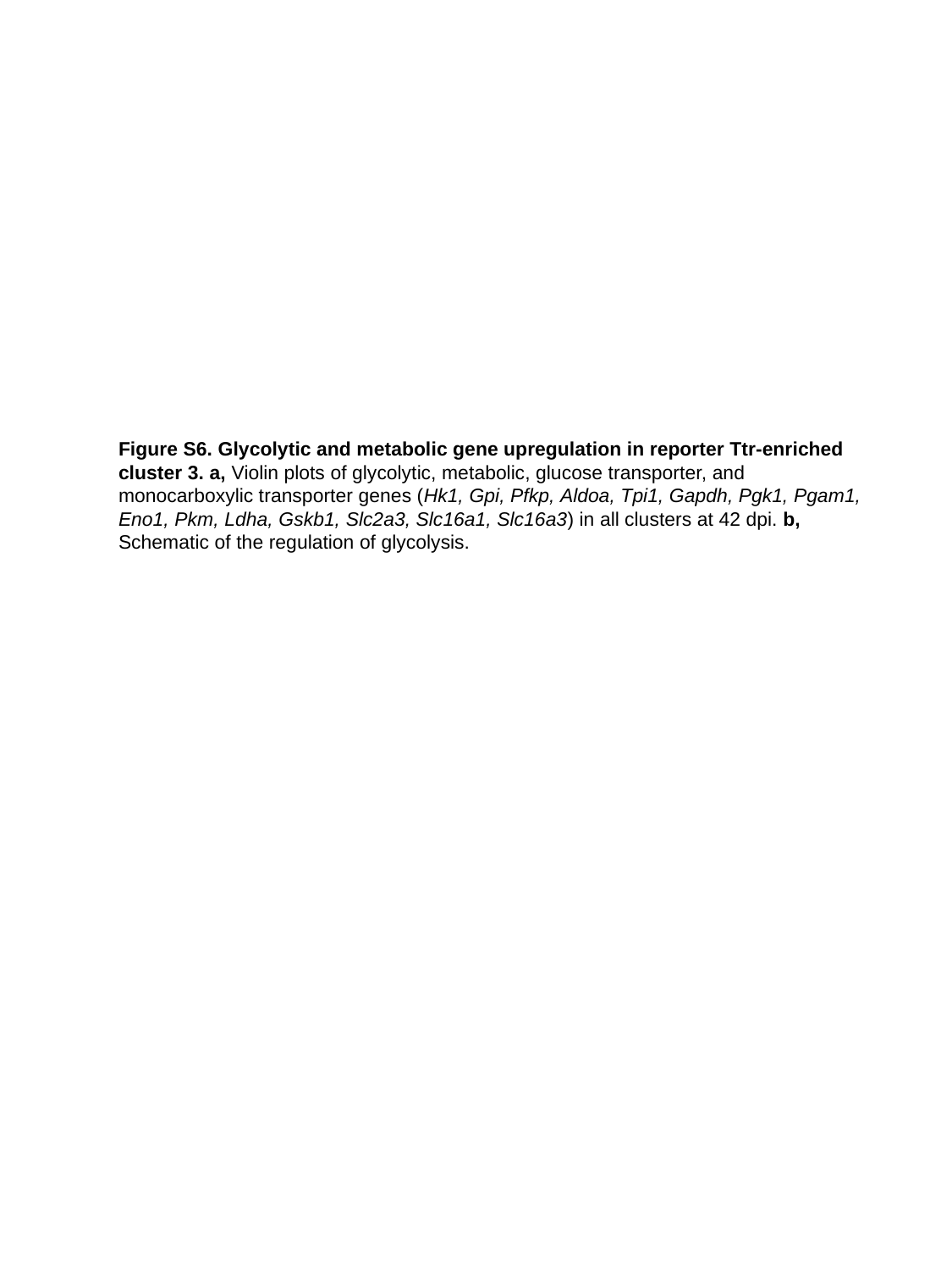

Figure S6. Glycolytic and metabolic gene upregulation in reporter Ttr-enriched cluster 3. a, Violin plots of glycolytic, metabolic, glucose transporter, and monocarboxylic transporter genes (Hk1, Gpi, Pfkp, Aldoa, Tpi1, Gapdh, Pgk1, Pgam1, Eno1, Pkm, Ldha, Gskb1, Slc2a3, Slc16a1, Slc16a3) in all clusters at 42 dpi. b, Schematic of the regulation of glycolysis.

## Slide 14
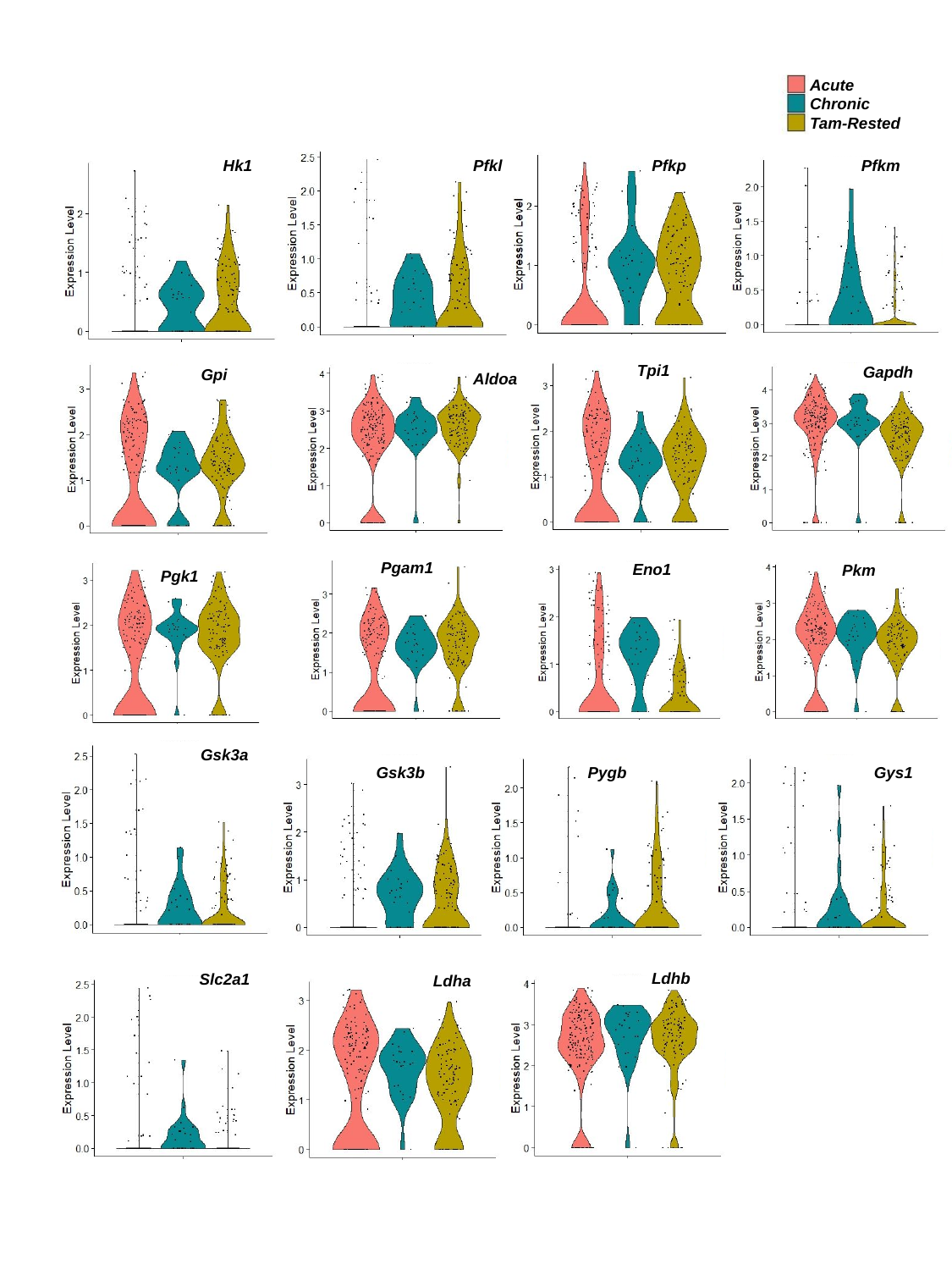

Acute
Chronic
Tam-Rested
Hk1
Pfkl
Pfkp
Pfkm
Tpi1
Gapdh
Gpi
Aldoa
Pgam1
Eno1
Pkm
Pgk1
Gsk3a
Gsk3b
Gys1
Pygb
Ldhb
Slc2a1
Ldha

## Slide 15
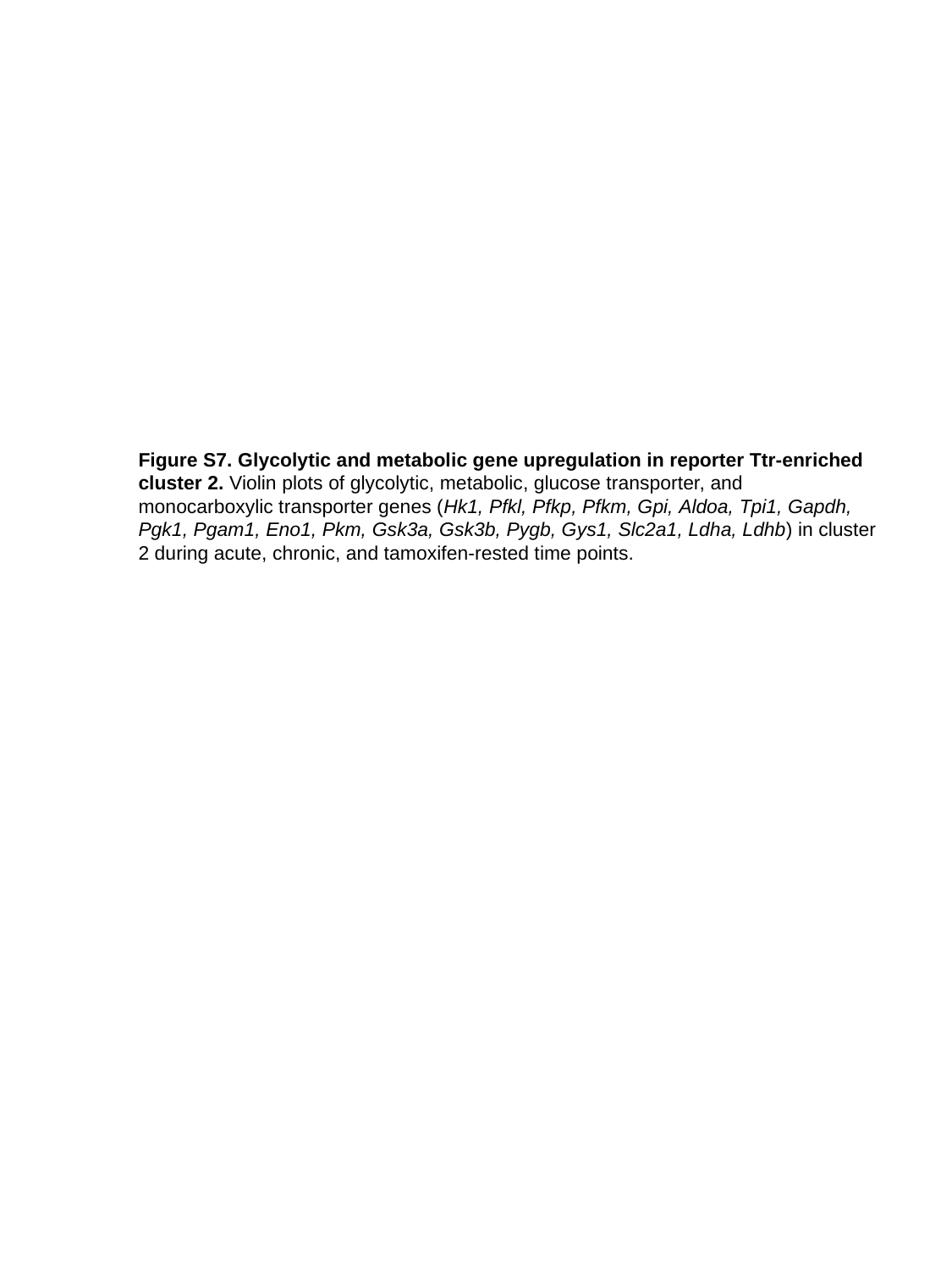

Figure S7. Glycolytic and metabolic gene upregulation in reporter Ttr-enriched cluster 2. Violin plots of glycolytic, metabolic, glucose transporter, and monocarboxylic transporter genes (Hk1, Pfkl, Pfkp, Pfkm, Gpi, Aldoa, Tpi1, Gapdh, Pgk1, Pgam1, Eno1, Pkm, Gsk3a, Gsk3b, Pygb, Gys1, Slc2a1, Ldha, Ldhb) in cluster 2 during acute, chronic, and tamoxifen-rested time points.

## Slide 16
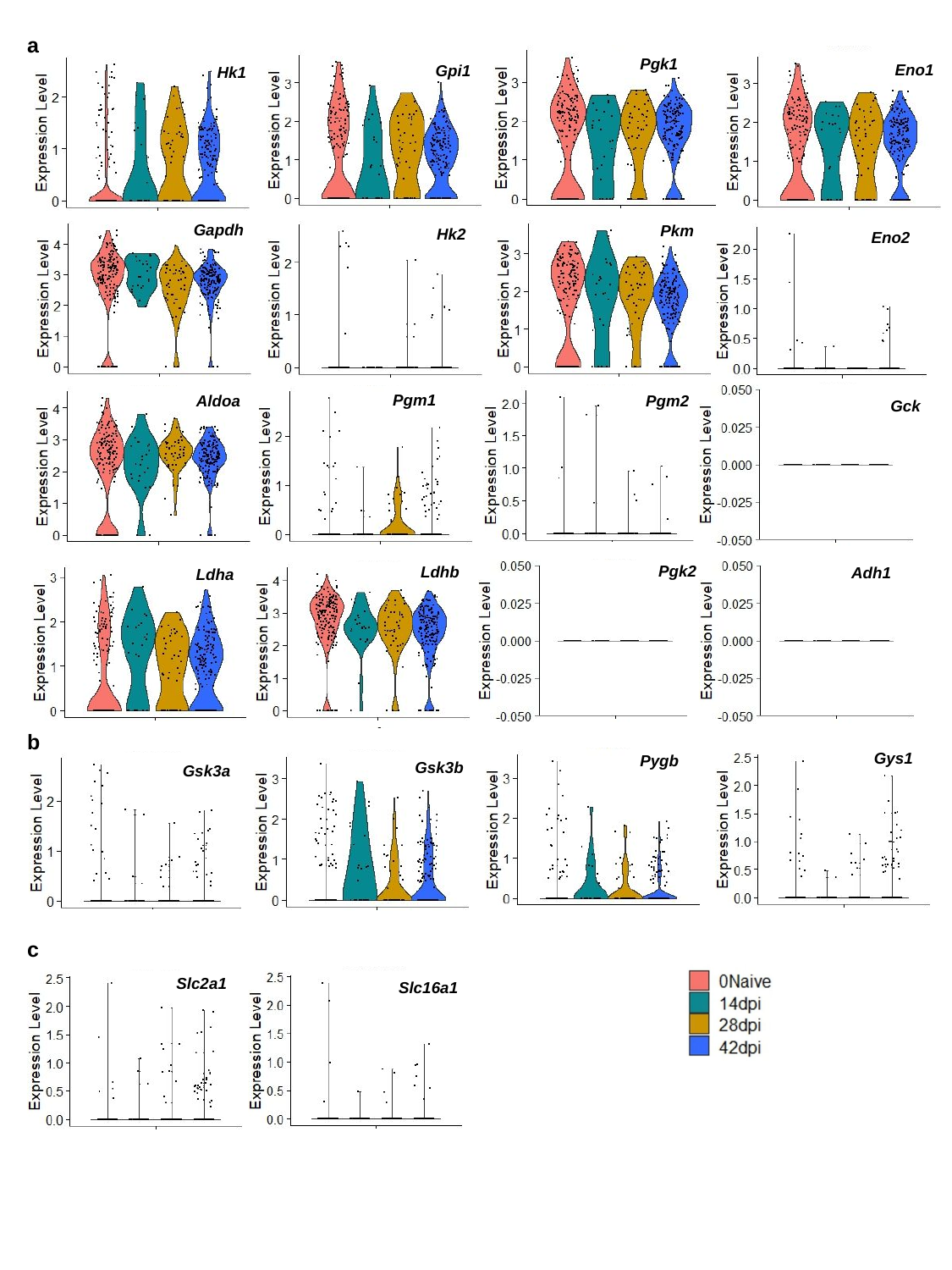

a
Pgk1
Eno1
Gpi1
Hk1
Gapdh
Pkm
Hk2
Eno2
Gck
Pgm1
Aldoa
Pgm2
Pgk2
Ldhb
Adh1
Ldha
b
Gys1
Pygb
Gsk3b
Gsk3a
c
Slc16a1
Slc2a1

## Slide 17
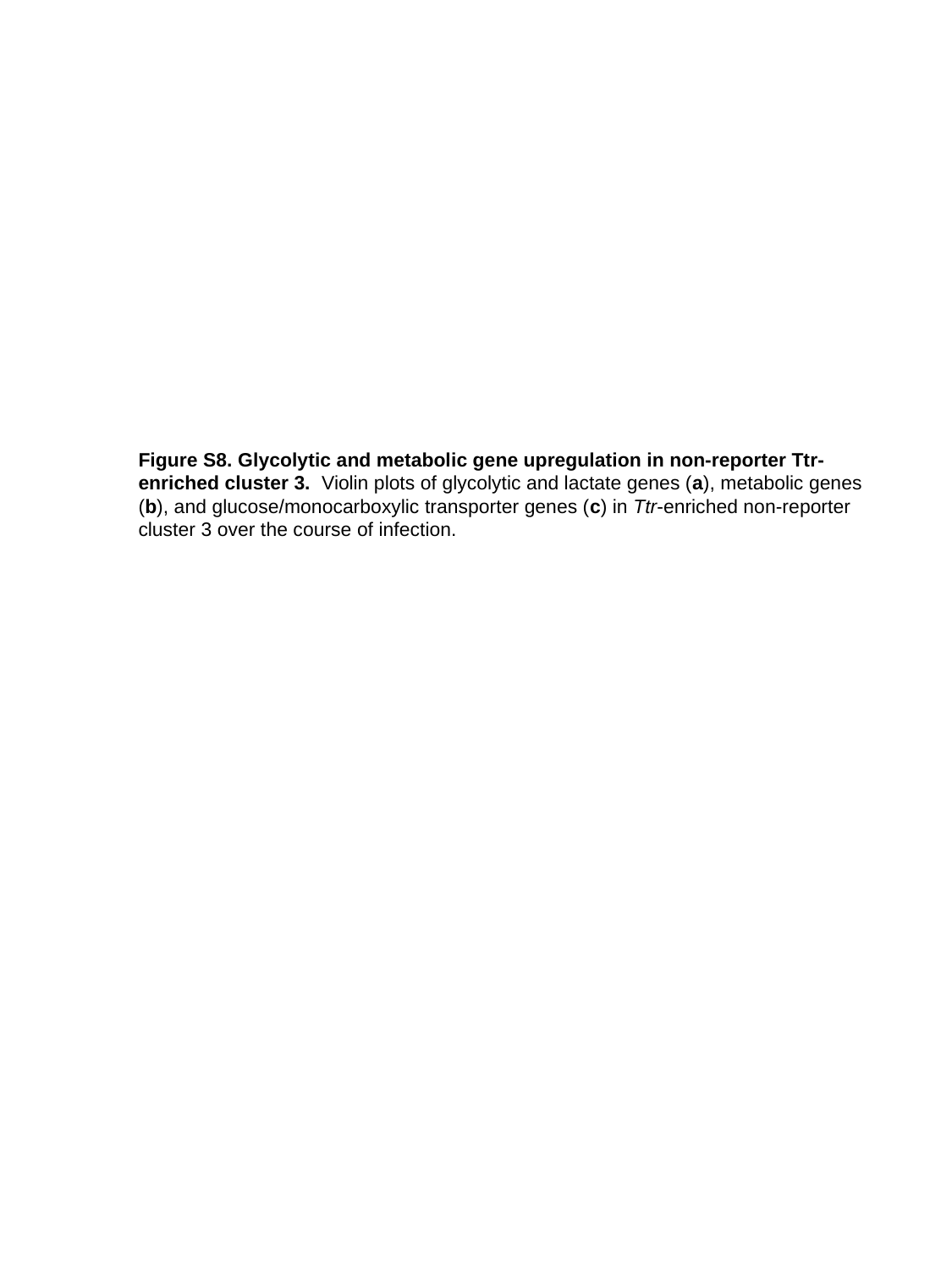

Figure S8. Glycolytic and metabolic gene upregulation in non-reporter Ttr-enriched cluster 3. Violin plots of glycolytic and lactate genes (a), metabolic genes (b), and glucose/monocarboxylic transporter genes (c) in Ttr-enriched non-reporter cluster 3 over the course of infection.

## Slide 18
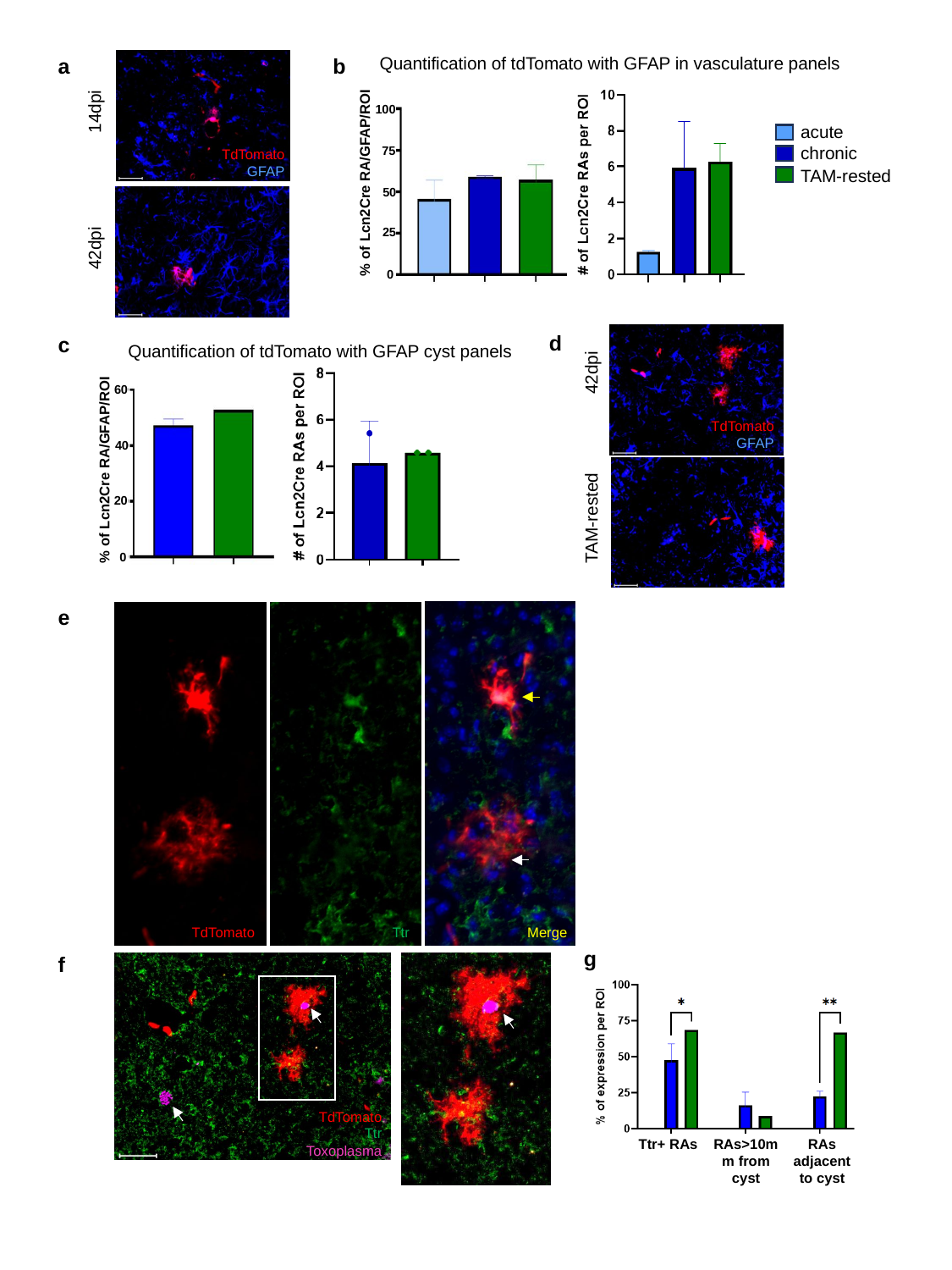

Quantification of tdTomato with GFAP in vasculature panels
a
b
14dpi
100
acute
chronic
75
TdTomato
GFAP
TAM-rested
% of Lcn2Cre RA/GFAP/ROI
50
42dpi
25
0
d
c
Quantification of tdTomato with GFAP cyst panels
42dpi
60
TdTomato
GFAP
40
% of Lcn2Cre RA/GFAP/ROI
20
TAM-rested
0
e
f
TdTomato
Ttr
Merge
g
f
TdTomato
Ttr
Toxoplasma
Ttr+ RAs
RAs>10mm from cyst
RAs adjacent to cyst
Merge

## Slide 19
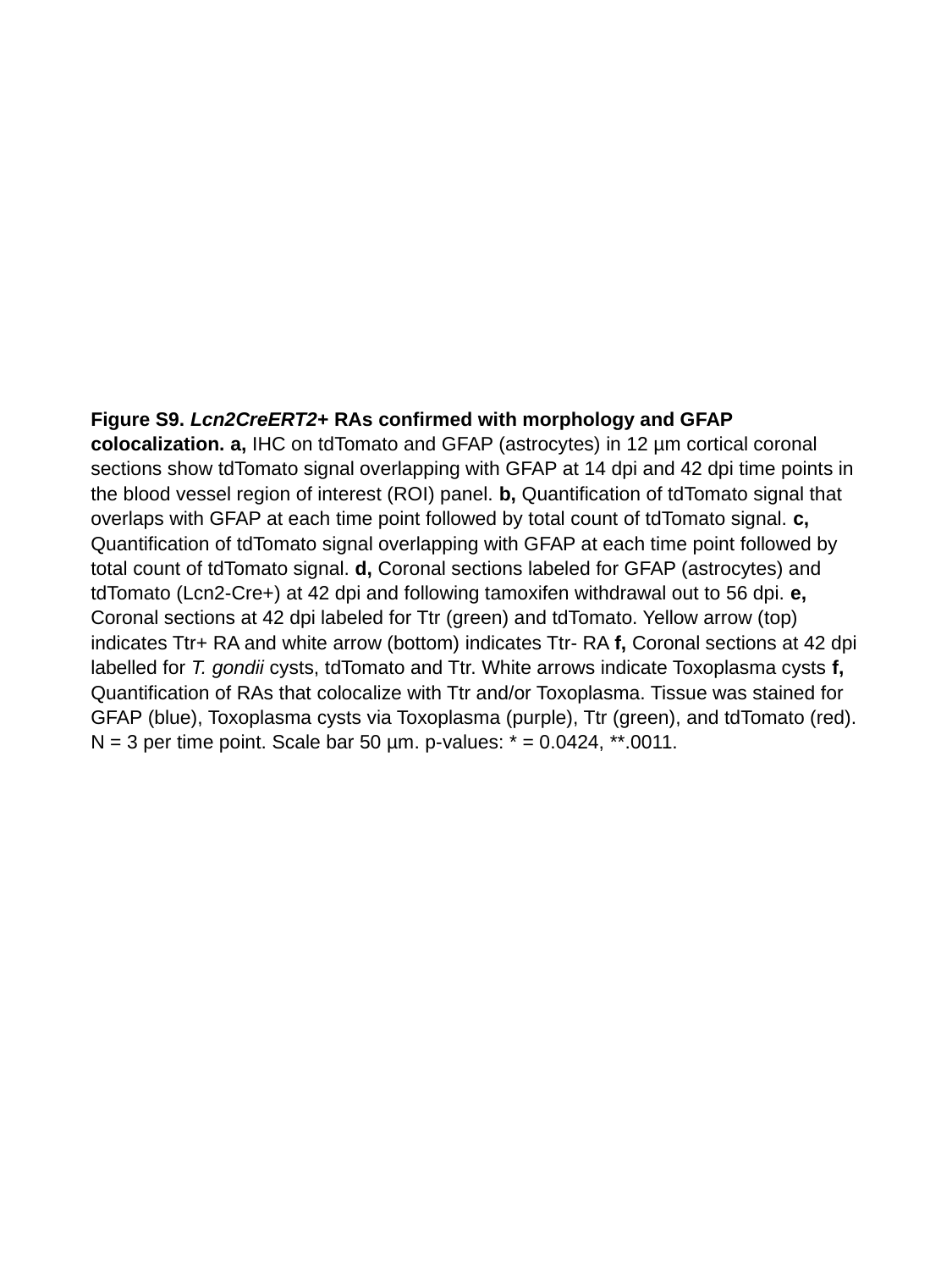

Figure S9. Lcn2CreERT2+ RAs confirmed with morphology and GFAP colocalization. a, IHC on tdTomato and GFAP (astrocytes) in 12 µm cortical coronal sections show tdTomato signal overlapping with GFAP at 14 dpi and 42 dpi time points in the blood vessel region of interest (ROI) panel. b, Quantification of tdTomato signal that overlaps with GFAP at each time point followed by total count of tdTomato signal. c, Quantification of tdTomato signal overlapping with GFAP at each time point followed by total count of tdTomato signal. d, Coronal sections labeled for GFAP (astrocytes) and tdTomato (Lcn2-Cre+) at 42 dpi and following tamoxifen withdrawal out to 56 dpi. e, Coronal sections at 42 dpi labeled for Ttr (green) and tdTomato. Yellow arrow (top) indicates Ttr+ RA and white arrow (bottom) indicates Ttr- RA f, Coronal sections at 42 dpi labelled for T. gondii cysts, tdTomato and Ttr. White arrows indicate Toxoplasma cysts f, Quantification of RAs that colocalize with Ttr and/or Toxoplasma. Tissue was stained for GFAP (blue), Toxoplasma cysts via Toxoplasma (purple), Ttr (green), and tdTomato (red). N = 3 per time point. Scale bar 50 µm. p-values: * = 0.0424, **.0011.

## Slide 20
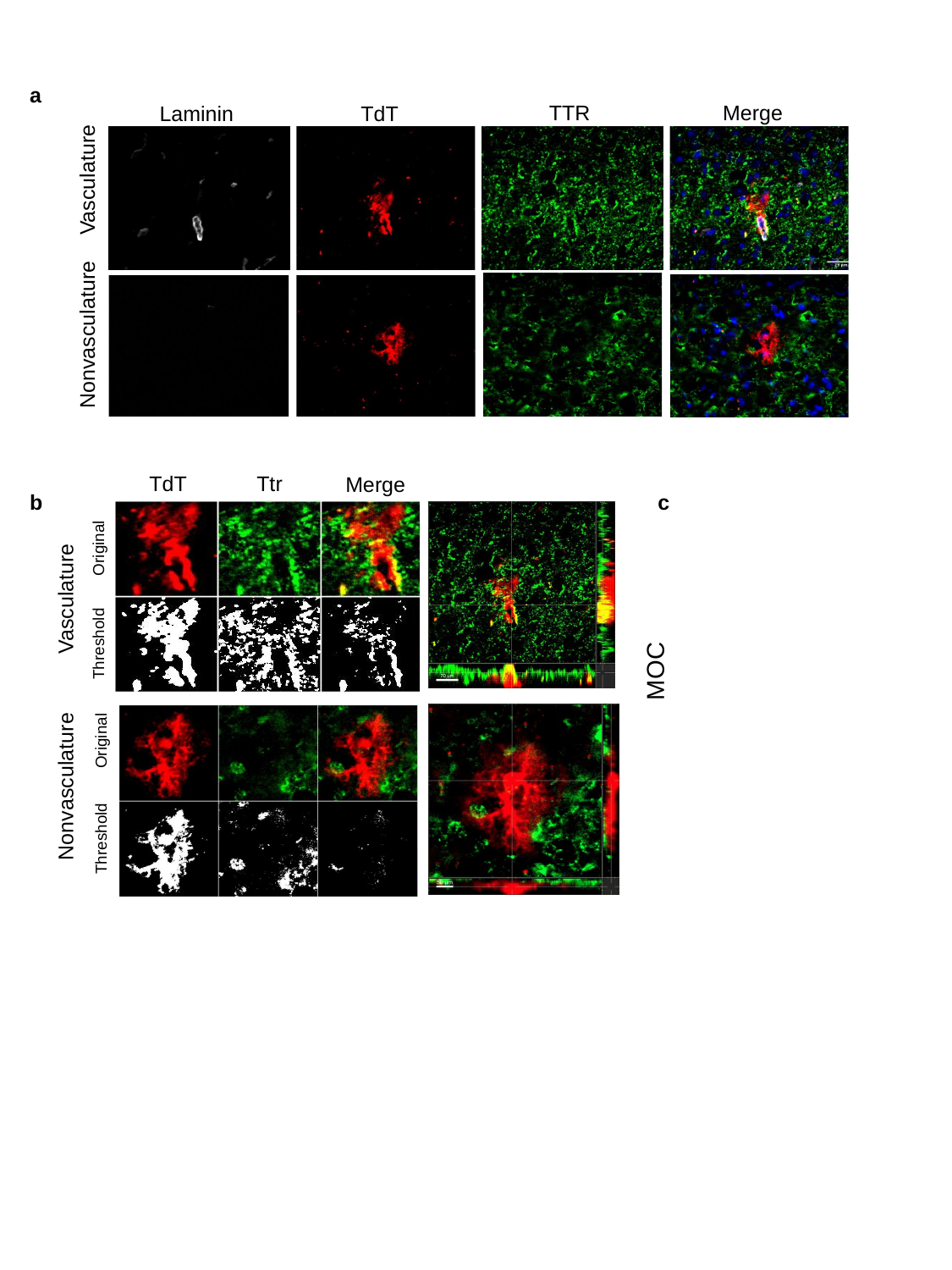

a
Merge
TTR
TdT
Laminin
Vasculature
Nonvasculature
TdT
Ttr
Merge
b
c
Original
Vasculature
Threshold
MOC
Original
Nonvasculature
Threshold

## Slide 21
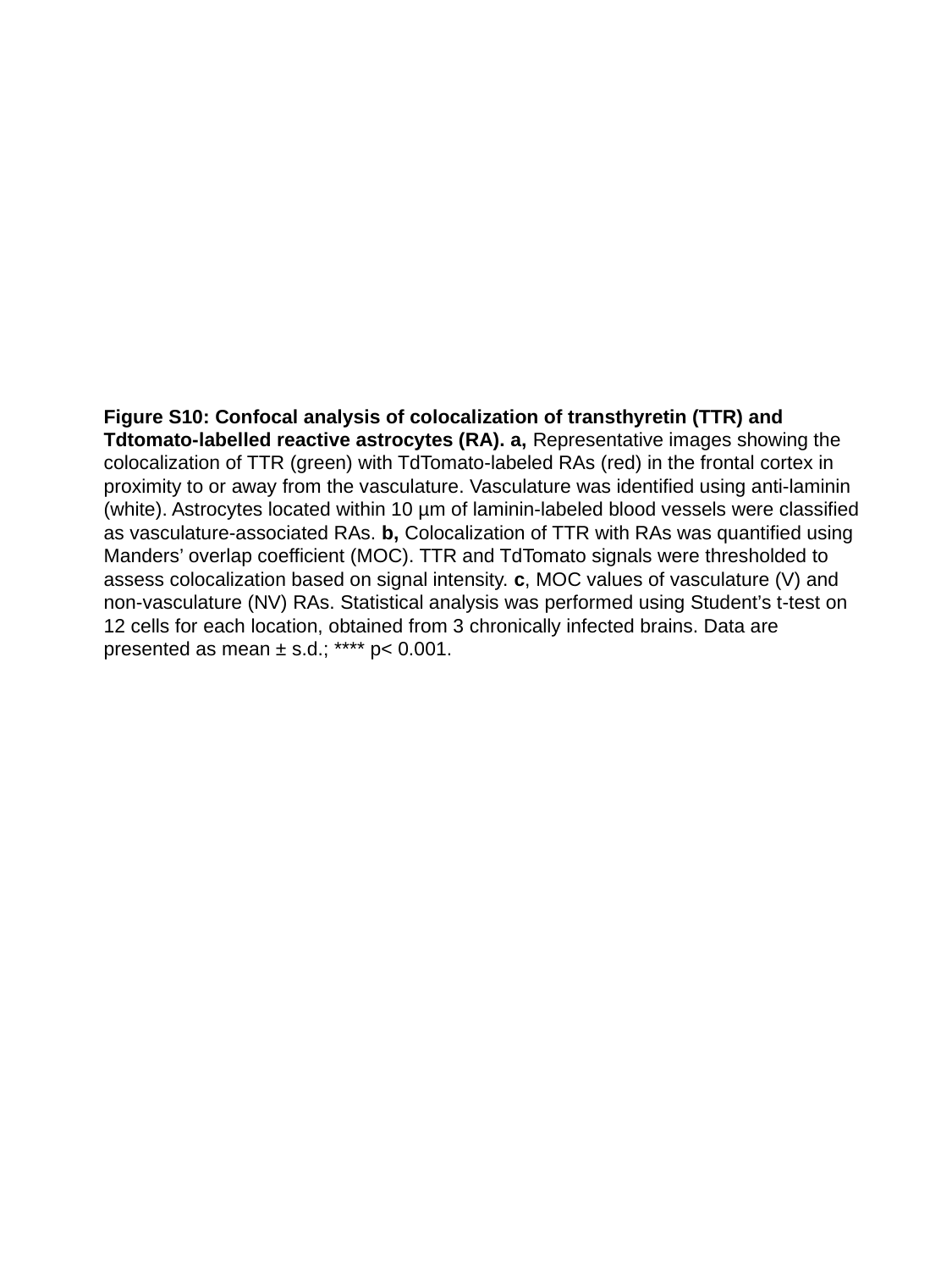

Figure S10: Confocal analysis of colocalization of transthyretin (TTR) and Tdtomato-labelled reactive astrocytes (RA). a, Representative images showing the colocalization of TTR (green) with TdTomato-labeled RAs (red) in the frontal cortex in proximity to or away from the vasculature. Vasculature was identified using anti-laminin (white). Astrocytes located within 10 µm of laminin-labeled blood vessels were classified as vasculature-associated RAs. b, Colocalization of TTR with RAs was quantified using Manders’ overlap coefficient (MOC). TTR and TdTomato signals were thresholded to assess colocalization based on signal intensity. c, MOC values of vasculature (V) and non-vasculature (NV) RAs. Statistical analysis was performed using Student’s t-test on 12 cells for each location, obtained from 3 chronically infected brains. Data are presented as mean ± s.d.; **** p< 0.001.

## Slide 22
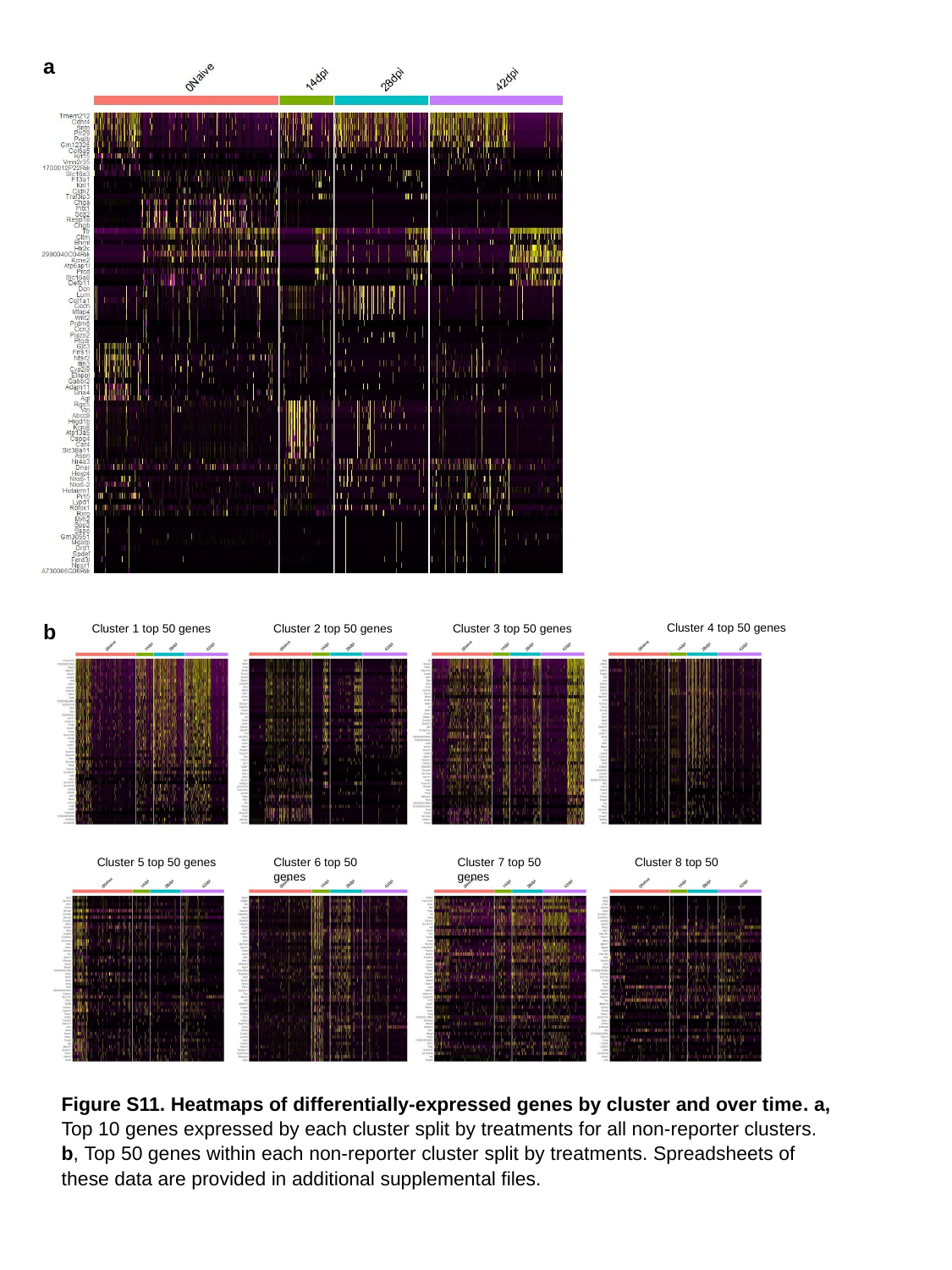

a
b
Cluster 4 top 50 genes
Cluster 2 top 50 genes
Cluster 3 top 50 genes
Cluster 1 top 50 genes
Cluster 8 top 50 genes
Cluster 7 top 50 genes
Cluster 5 top 50 genes
Cluster 6 top 50 genes
Figure S11. Heatmaps of differentially-expressed genes by cluster and over time. a, Top 10 genes expressed by each cluster split by treatments for all non-reporter clusters. b, Top 50 genes within each non-reporter cluster split by treatments. Spreadsheets of these data are provided in additional supplemental files.

## Slide 23
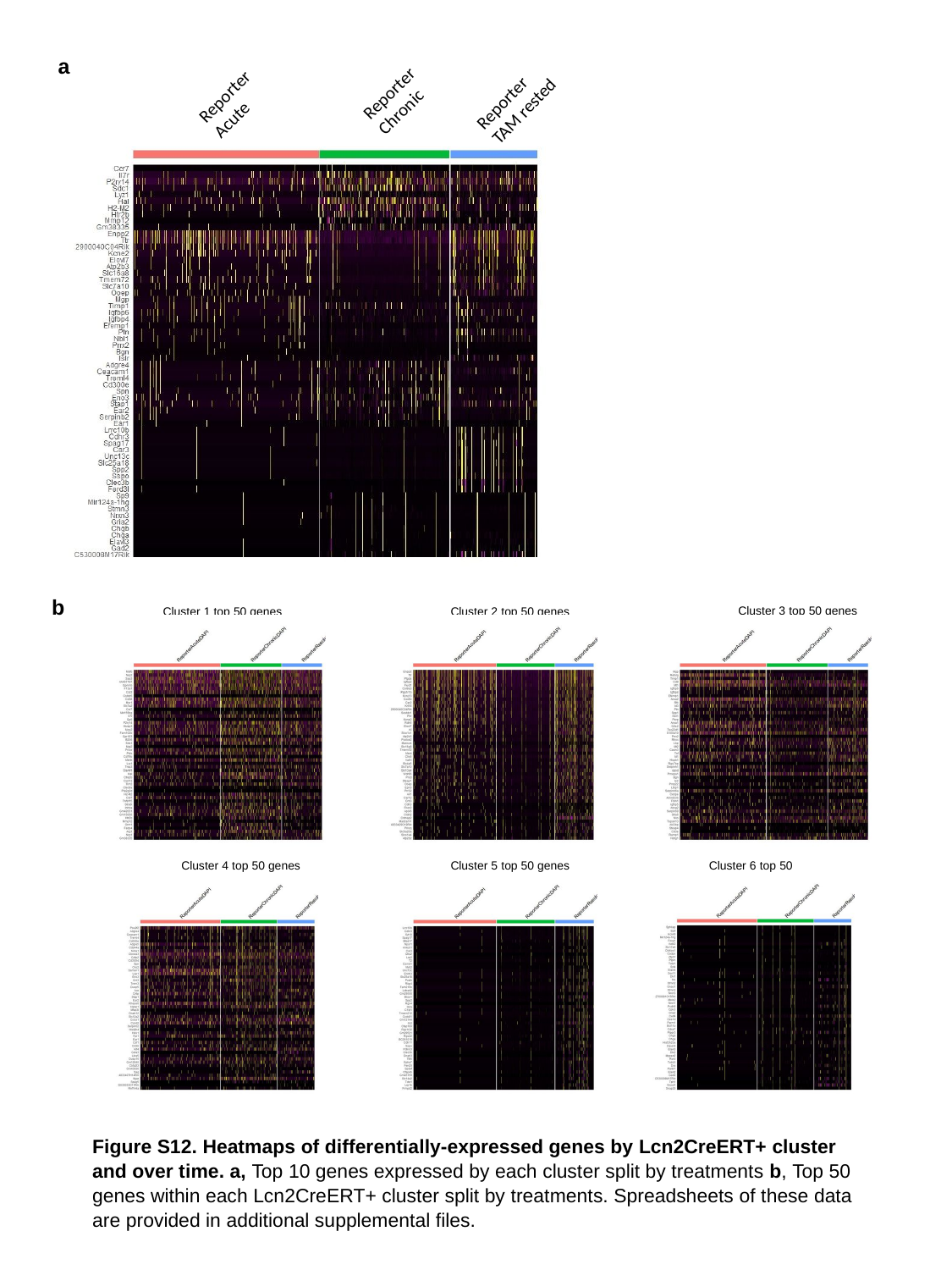

Reporter TAM rested
Reporter Chronic
Reporter Acute
a
b
Cluster 3 top 50 genes
Cluster 1 top 50 genes
Cluster 2 top 50 genes
Cluster 5 top 50 genes
Cluster 6 top 50 genes
Cluster 4 top 50 genes
Figure S12. Heatmaps of differentially-expressed genes by Lcn2CreERT+ cluster and over time. a, Top 10 genes expressed by each cluster split by treatments b, Top 50 genes within each Lcn2CreERT+ cluster split by treatments. Spreadsheets of these data are provided in additional supplemental files.
